# Self‐organizing cicada choruses respond to the local sound and light environment

**DOI:** 10.1002/ece3.6213

**Published:** 2020-04-20

**Authors:** Lawrence W. Sheppard, Brandon Mechtley, Jonathan A. Walter, Daniel C. Reuman

**Affiliations:** ^1^ Department of Ecology and Evolutionary Biology and Kansas Biological Survey University of Kansas Lawrence KS USA; ^2^ School of Arts, Media and Engineering Arizona State University Tempe AZ USA; ^3^ Department of Environmental Sciences University of Virginia Charlottesville VA USA; ^4^ Laboratory of Populations Rockefeller University New York NY USA

**Keywords:** cicada, citizen science, insect chorus, synchrony, wavelet

## Abstract

1. Periodical cicadas exhibit an extraordinary capacity for self‐organizing spatially synchronous breeding behavior. The regular emergence of periodical cicada broods across the United States is a phenomenon of longstanding public and scientific interest, as the cicadas of each brood emerge in huge numbers and briefly dominate their ecosystem. During the emergence, the 17‐year periodical cicada species *Magicicada cassini* is found to form synchronized choruses, and we investigated their chorusing behavior from the standpoint of spatial synchrony.
2. Cicada choruses were observed to form in trees, calling regularly every five seconds. In order to determine the limits of this self‐organizing behavior, we set out to quantify the spatial synchronization between cicada call choruses in different trees, and how and why this varies in space and time.
3. We performed 20 simultaneous recordings in Clinton State Park, Kansas, in June 2015 (Brood IV), with a team of citizen‐science volunteers using consumer equipment (smartphones). We use a wavelet approach to show in detail how spatially synchronous, self‐organized chorusing varies across the forest.
4. We show how conditions that increase the strength of audio interactions between cicadas also increase the spatial synchrony of their chorusing. Higher forest canopy light levels increase cicada activity, corresponding to faster and higher‐amplitude chorus cycling and to greater synchrony of cycles across space. We implemented a relaxation‐oscillator‐ensemble model of interacting cicadas, finding that a tendency to call more often, driven by light levels, results in all these effects.
5. Results demonstrate how the capacity to self‐organize in ecology depends sensitively on environmental conditions. Spatially correlated modulation of cycling rate by an external driver can also promote self‐organization of phase synchrony.

## Graphical abstract

The spatial synchrony of cicada choruses was investigated using data recorded by volunteers with smartphones. Self‐organized phase synchrony of call cycling is found to extend through the forest. Changes in canopy illumination are correlated with changes in cicada cycling rate and degree of phase synchrony. Photo Credit: Prof. Ben Sikes, KBS, University of Kansas.

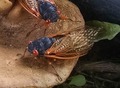

## 1 INTRODUCTION

In ecology, spatial synchrony is often observed in fluctuations of populations produced by the action of environmental changes, the activity of predators, or other synchronous drivers, without the necessity of direct interactions between the populations in space. We refer to this first kind of spatial synchrony as type 1. However, synchronous fluctuations may also arise as a result of phase synchronization between self‐sustaining oscillations (Pikovsky, Rosenblum, & Kurths, 2001; Strogatz, 2000; Strogatz & Stewart, 1993). In this case, the synchronization is dependent on interactions between the oscillators rather than (or in addition to) an external organizing force. Interactions bring the oscillators “into phase with each other,” that is, all oscillators reach a maximum at the same point in time, and a minimum at the same point in time. We refer to this second, self‐organizing type of spatial synchrony as type 2. Periodical cicadas famously exhibit spatially synchronous emergence across space, for the purpose of mating (Alexander & Moore, 1962; Gerhard, 1923; Marlatt, 1923; Myers, 1929; Williams & Simon, 1995). In *Magicicada cassini*, which emerges every 17 years (and in *M. tredecassini*, a closely related species that emerges every 13 years), the males are also observed to chorus with periodical increases in volume, punctuated by flight activity (Alexander & Moore, 1962; see supplementary information for a clip of cicada sound from a single tree). Choruses last from days to weeks (Williams & Smith, 1991). For both emergence and cyclic chorusing, the collective periodic behavior can be described by a phase variable at each location, and this phase variable has the potential to be permanently shifted forward or backward by perturbations associated with the phase at neighboring locations. These are the necessary conditions for self‐organizing synchronous behavior (Strogatz, 2000; type 2 synchrony). Thus, the same interactions between individuals that lead to periodic chorusing behavior at a location could lead to spatial synchrony between locations. The purpose of this study was to examine the spatial characteristics of synchrony of chorusing sound volume oscillations in *M. cassini*.

A wide range of arthropods use sound to communicate and attract mates, from the spider *Hygrolycosa rubrofasciata* (Kotiaho, Alatalo, Mappes, & Parri, 2004) to the fly *Drosophilia* (Clyne & Miesenbock, 2008). Not all calling insects form choruses: pairwise calling synchrony between males of the coneheaded katydid, *Neoconocephalus nebrascensis*, is observed in male–male interactions, where the leading katydid is dominant (Meixner & Shaw, 1986). Competing male *Gryllus integer* field crickets may either call to attract mates, or search for mates in their vicinity, with both calling and searching strategies being evolutionarily viable (Cade & Cade, 1992). In the cicada *Cystosoma saunders*, among other behavioral strategies, males occurred in pairs, trios, and other multiples in bushes more frequently than would be expected in random distributions. Such males were found to attract more females per capita than did isolated males (Doolan & Nally, 1981). This may represent a minimal form of the large‐scale chorusing found in *Magicicada* and other species. Past work on insect choruses, specifically, has focused mostly on Orthoptera (particularly katydids) and cicadas. Studies on cycling synchrony in *M. cassini* and *M. tredecassini* cicadas have been rendered uncommon in part because of the long emergence periods of these species.

Chorusing behavior in insects may provide direct fitness benefits to individuals for a variety of reasons (Greenfield, 1994a; Greenfield, Tourtellot, & Snedden, 1997; Hartbauer & Römer, 2016). Greenfield (2015) reviews some possible advantages of chorusing (e.g., providing a “beacon” to attract mates, avoiding predation) while also discussing the ways in which insects may modify the phase of their calling cycle in response to the calls of others. As in the visual displays of the synchronizing firefly *Pteroptyx malac* (Buck & Buck, 1978), calling synchronization may increase the “signal‐to‐noise ratio” of the acoustic signal via both “augmentation of peak intensity and intermittency of signal.” Female *Photinuscarolinus* fireflies are found responsive to synchronous flashes only (Moiseff & Copeland, 2010), perhaps explaining the tendency to widespread spatial synchronization of male firefly displays. Even if synchrony is not intrinsically attractive to females, synchronized calls may be clearer. Grobe, Rothbart, Hanschke, and Hennig (2012) describe the various features of a calling pattern that females use to discriminate between males of the field cricket *Gryllus bimaculatus*, and Balakrishnan, Helversen, and Helversen (2001) describe how the pattern of calling and pausing is crucial to pair formation in the duetting grasshopper *Chorthippus biguttulus*. For some insects, rather than merely maintaining high activity, chorusing insects must maintain exact timing relationships between calls if temporal call structure is to be perceptible to females. Deily and Schul (2009) suggest that *N. nebrascensis* engages in cooperative chorusing as the complexity of the pulse pattern required for species recognition would otherwise be obscured. In addition to transmitting an attractive and clear signal for females, synchrony may benefit males by confusing predators. For example, by forming a chorus, the moth *Achroia grisella* wastes less time avoiding predators (Brunel‐Pons, Alem, & Greenfield, 2011).

Call synchrony can also arise more as a side effect of competitive interactions between males. Greenfield (1994a) introduced the possibility that when calls overlap, the first to call may effectively “jam” the signals of other callers from the point of view of a receptive female, leading to synchronized calling as a byproduct. The advantage of calling first is found to be widespread where a receptive mate is oriented by sound, including under chorus conditions: for example, Berg and Greenfield (2005) describe the katydid *Ephippiger ephippiger*, a species in which females prefer leading calls, with males in neighborhoods with more overall singing also being preferred (see also Snedden & Greenfield, 1998). Although Hartbauer, Haitzinger, Kainz, and Römer (2014) examined the potential advantages of a chorus in increasing peak sound volume and stabilizing calling rate in the katydid *Mecopoda elongata*, they also examined the competitive strategies that can give rise to choruses, in a series of experimental manipulations. Fertschai, Stradner, and Römer (2007) suggest that leading calls may be less attractive to females than sufficiently loud subsequent calls in *M. elongata*; thus, when one male calls, others may be triggered to outcompete it, resulting in a chorusing pattern. The model of *M. elongata* in Hartbauer (2008) simulates the chorus as a set of interacting coupled oscillators and reproduces the chorus synchrony. For chorusing insects generally, the tendency of male insects of a given species to attempt to either lead (Greenfield, 1994b) or simply reinforce the ambient calling sound may depend on the preferences of females (Greenfield, Marin‐Cudraz, & Party, 2017; Greenfield & Roizen, 1993) of the species.

Finally, calling and chorusing behavior can be modified by the environment, a fact that can complicate studies of the evolutionary reasons for chorusing. For example, females of the field cricket, *Gryllus rubens*, exhibit a preference for male calls of an appropriate periodicity. But, the calling period of the male is itself modulated by increasing temperature, which can reduce the attractiveness of the males (Doherty & Callos, 1991).

The literature on other species suggests hypotheses on what mechanisms may cause chorus synchrony in *M. cassini*, but, because of their large numbers, the acoustic landscape produced by *M. cassini* differs markedly from that of many prior model species, and therefore, in our view, a deeper understanding of the reasons for synchronous chorusing in *M. cassini* and *M.tredecassini* will likely require a distinct approach. As was the case for the moth *Achroia grisella* (Brunel‐Pons etal., 2011), it is possible, a priori, that the synchronous chorusing behavior of *M. cassini* may serve the purpose of confusing predators. Other possible explanations include the increased attractant effect on females of many male cicadas calling together (cooperative strategy, as for, e.g., *Cystosoma saunders*, Doolan & Nally, 1981), or that synchrony is a side effect of cicadas trying to call as often as possible and ahead of their companions (competitive strategy, as may have been the case for, e.g., *M. elongata*, Hartbauer et al., 2014). Mate choice in *Magicicada* includes visual and auditory cues (Cooley, 1999), suggesting that males may engage in cooperative chorusing in order to draw females to an area, followed by more individualistic behaviors to accomplish mating itself (Cooley & Marshall, 2001), but Cooley and Marshall (2004) find no evidence of female selectivity in *M. septendecim*. Female song preferences may at least help reduce interspecies mating attempts (Marshall & Cooley, 2000).

Ultimately, the research done so far on phase‐synchronized chorusing in *M. cassini* and *M. tredecassini* is too limited to definitively test alternative hypotheses about the behavior. The acoustic landscape generated by *M. cassini* is markedly distinct from some of the prior studies in which mechanisms could be distinguished. Where it is possible to record acoustic signals coming from individuals or small groups of animals (e.g., *Cystosoma saunders*, Doolan & Nally, 1981; *Sorapagus catalaunicus* and *Ephippiger diurnus*, Greenfield et al., 2017), such recordings can be used to distinguish mechanisms. *M. cassini*, however, are so abundant and prone to movement, and their sound is so omnipresent during an emergence that recordings of individual cicadas participating in cyclic chorusing in a natural setting may be impractical. It would additionally be difficult to measure fitness advantages or disadvantages that individual cicadas may experience as a result of variation in their synchronous chorusing behavior. In our view, research should proceed instead to measure spatiotemporal statistical characteristics of the acoustic environment generated by cicadas, and then to use this information, together with mechanistic modeling, to build toward inferences of how synchronous chorusing emerges and what are its evolutionary mechanisms. Inferences can be based on which mechanistic hypotheses, when built into a model, can reproduce key statistical aspects of the observed acoustic signature of *M. cassini*. We here take the first steps toward this research goal by characterizing new aspects of the acoustic environment produced by *M. cassini*. We also develop an initial model of *M. cassini* chorusing. Though our model is not yet sufficiently developed to distinguish evolutionary mechanisms for synchronous chorusing in the species, we see it as an initial step which can facilitate the later development of such a model. The spatiotemporal pattern of synchrony is complex and therefore may be an effective tool for discriminating hypotheses—incorrect models are unlikely to accidentally reproduce details.

This study addresses the brood IV (as numbered by Marlatt, 1923 and Simon, 1988) cicada emergence in June 2015. Each numbered cicada brood includes three species which are synchronized in their emergence period (Alexander & Moore, 1962). In the case of the 17‐year cicadas, these are *M. septendecim* (Linnaeus, 1758), *M. cassini* (Fisher, 1851), and *M. septendecula* (Alexander & Moore, 1962). Each has a counterpart species in the 13‐year periodical broods (e.g., the counterpart of *M. cassini* is *M. tredecassini*). *M. cassini* is abundant in Northeastern Kansas (see maps in Alexander & Moore, 1962), and its distinctive cyclic chorusing behavior was clearly apparent in our study area. See Appendix S1 for more background on *M. cassini* emergence and behavior.

We took advantage of the widespread availability of accurate and portable digital consumer sound recording devices in 2015 (smartphones, not generally available in 1998, the last emergence) to make simultaneous spatially distributed recordings of cicada chorusing over an afternoon. Since a range of devices including different hardware and software were in use, and all devices were placed on the ground with the forest canopy (and cicadas) at variable heights above, it was not possible to compare the absolute volume of sound at different locations. Instead, we examined the variability in sound volume of each recorder, as recorders were synchronized and each recorder measures time accurately and comparably. The most notable feature of this variability in local cicada sound volume is the periodic five‐second cycling of the chorus, and the relative timing of the peaks and troughs in these cycles can be compared accurately between sites to definitively demonstrate intersite type 2 spatial synchrony in volume, irrespective of the incomparable absolute volume levels recorded by the devices. The nature of spatial synchrony of *M. cassini* chorus volume oscillations is the statistical aspect of the acoustic field that we investigate.

Where physical factors alter the behavior of individual cicadas in a spatially synchronized way, there exists the potential for changes in their collective behavior as a result, including the phase synchronization of chorusing cycles (which are not in themselves driven by changes in the environment). *Magicicada* calling activity is dependent on illumination (Myers, 1929, p. 206–207) and temperature (George, 1920). Female periodical cicadas are observed to use light cues to select oviposition sites (Yang, 2006). This motivates an investigation of the effects of sunlight on spatial synchrony of calling: the degree of type 1 synchronization of calling *activity* may determine the degree of type 2 synchronization of call‐volume oscillations. We will investigate the effect on synchrony of a change in cycle rate induced by light levels. Such a change was observed to have significant effects on the chorusing of other insects, for example, the field cricket *Gryllus rubens* (Doherty & Callos, 1991).

Recordings were made in Clinton State Park, Kansas, at the western edge of the range for *M. cassini* (Alexander & Moore, 1962; Cooley et al., 2009; Marshall, 2001; Marshall, Cooley, & Simon, 2003). We also recorded canopy light levels with a single upward‐facing camera, to test the effect of light on cicada behavior. In order to trace the dynamic behavior of the cycling cicada choruses, we applied a wavelet transform to each sound volume time series. The Morlet wavelet transform provides a natural way to examine periodical behavior and synchrony, as it yields a phase and amplitude of oscillation for each point in time for every timescale examined (Addison, 2002). Thus, we could follow how the characteristic amplitude and rate of volume oscillation change through time by tracing the movement through time of the peak in the wavelet transform magnitude; and we could examine how spatial synchrony changed by comparing transforms from different recording locations.

A spatially isolated *M. cassini* chorus demonstrates clear calling synchrony with a characteristic five‐second rise and fall in volume: a cycle. The phase of a cycle is an angle variable that describes its evolution through time; the chorus phase may be considered to start at zero when the volume is highest, reach a phase of *π* radians at the volume minimum, and a phase of 2*π* radians at the next maximum, at which point it returns to zero. The collective behavior of the chorusing cicadas is apparent in a single recording of a chorus, and a priori a chorus may extend for some distance spatially, leading to spatial synchrony of cicada cycles that might be recorded at different locations with independent devices. However, how far sound cycle phase synchrony can extend through a continuous forest canopy is impossible for a single observer to discern. We wished to determine the distance over which synchrony in volume fluctuations was maintained, by simultaneous recording of spatially separated sites. Like localized chorusing, such spatial synchrony is an emergent phenomenon resulting from the responsiveness of individual insects to sound stimulus. Long‐distance chorus coordination must be subject to additional effects of sound attenuation and delay. We also investigated environmental factors affecting the tendency to synchronous cycling, addressing the hypothesis that ambient light levels affect the degree ofsynchronization of male cicadas during chorusing. After finding empirical evidence that increases in calling synchrony correspond to increases in ambient light levels, we used arelaxation‐oscillator model to show how increases in calling synchrony can be attributed mechanistically to the increases in the calling rate of individual cicadas under stronger illumination. We view our efforts to characterize the spatiotemporal acoustic signal associated with *M. cassini* and to formulate initial models which can reproduce important aspects of this signal as first steps of an approach to understanding the reasons for chorusing in this species that could be practical in light of the particular ecological characteristics of the species.

## 2 MATERIALS AND METHODS

### 2.1 Data

We sampled chorus volume in the forest at Clinton State Park in the afternoon of 18 June 2015, where periodic fluctuations in cicada volume could be heard. Volunteers brought smartphones and other digital recording devices to the site, activated recording, and all recorded a primary reference event (a balloon pop, t = 0) to ensure exact timing. Recorders were then dispersed to approximately evenly spaced locations along the woodland trails. These locations were GPS tagged with multiple GPS devices. The maximum distance between recording locations at the main site was approximately 436 m, between locations 8 and 15. One simultaneous recording was also made about 3 km away, for an off‐site reference.

After all recording devices were in place at their recording locations, the volunteers left the woods and we recorded approximately 100 min of undisturbed simultaneous audio on all the devices. External disturbances not associated with our volunteers (wind, bird calls, and a passing helicopter) can be heard on the recordings. Devices were collected and together recorded a secondary reference event (another balloon pop) before being switched off. We used the reference events to ensure simultaneity, and we truncated the audio recordings to remove all data before the last device was placed and after the first device was recovered. Details are provided in Appendix S2.

One of the recording devices was an Apple iPad (location 5) which was left recording video of the forest canopy/overcast sky simultaneously with audio. The video frame data of the forest canopy/sky were truncated in the same way, and each frame was reduced to a single mean brightness value by summing the values of all pixels. A five‐minute moving‐average brightness profile time series was used for analysis. Details are provided in Appendix S3.

### 2.2 Statistics

**FIGURE 1:**
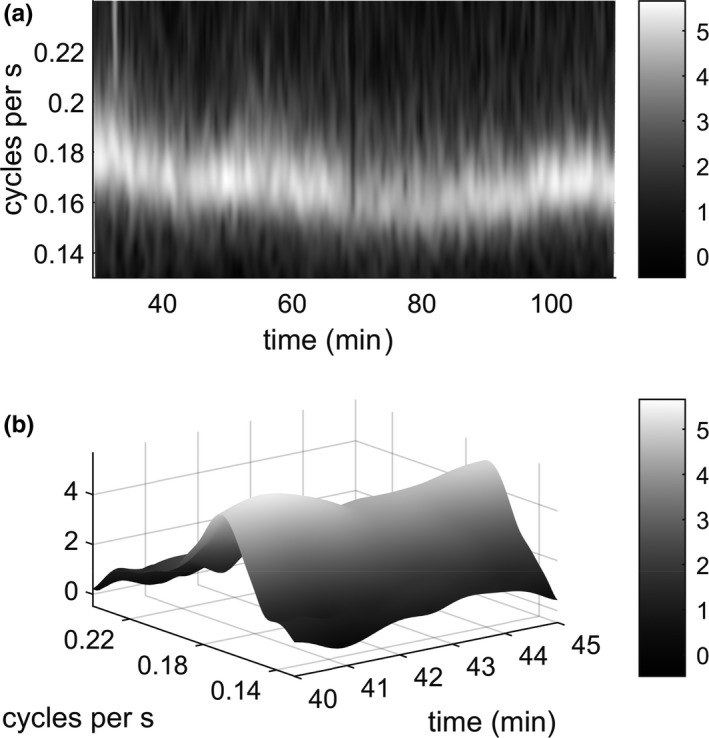
Changing frequency of fluctuations in sound volume in the forest. (a) Log of average of transform energies (squared modulus of the transforms), where each transform was first scaled to have total time‐averaged energy 1 within a frequency range of 1/8 to 1/3 cycles per second. Time 0 is a primary reference event used to temporally match recordings (see Methods: Data). Time range shown here is the interval within which all sensors were active and undisturbed, beginning 3:54 p.m. local time. (b) A five‐minute segment of the above plot, presented in 3d to make the local maximum (at around 0.17 cycles per second) clearly visible. The cycling frequency associated with this feature is presented in Figure 3a as it varies over the whole period of measurement

Sound spectral characteristics of cicada calls varied somewhat by recording location (Figure S1). In order to trace the dynamic behavior of the cycling cicada choruses, we calculated sound volume time series for each recording with 0.1‐s resolution. We Fourier‐transformed the values in each 0.1‐s segment, filtered them, and took the total squared amplitude of the values in the filtered Fourier transform as our measure of (relative) sound volume. We applied a wavelet transform to each sound volume time series, allowing us to trace the phase and magnitude of the observed 5‐s cycle in cicada volume over time. We filtered recordings to isolate cicada‐related acoustic activity, using a “matched” filter to extract the volume of cicada chorusing in each recording at each point in time (Appendix S2). We then worked with temporal variability in the volume of cicadas in all analyses below. Complex Morlet wavelet transforms identified the regular cycling in cicada volume at each location, with a variable period of approximately 5 s (0.2 Hz, Figures S2–S21). We identified the spectral peak in the average (over locations) transform energy of cicada volume fluctuations to determine the average cycling period of the whole forest at each point in time (Figure 1). The changes in this peak position correspond to faster and slower cycling. For comparability, at each point in time we compared wavelet phases and amplitudes from different locations drawn from their transforms at this whole‐forest period. Details are provided in Appendix S2.

We used several methods to identify spatial synchrony in the phases drawn from the 20 locations, all based on wavelet transform values, at the cycling frequency of the forest, extracted from each location at each point in time. First, these complex time series of length *T* were checked pairwise for systematic associations between phases, $\phi_{j,t}$ , $\phi_{k,t}$ , over time, *t*, using phase coherence, which is the magnitude of $\frac{1}{T}\sum_{t}e^{i\left(\phi_{j,t}-\phi_{k,t}\right)}$ (Sheppard, Stefanovska, & McClintock, 2012). The typical phase difference in recording locations *j* and *k* is the phase of this quantity. Phase coherence is low when two sites do not agree on a common cycling frequency, or when they have the same mean cycling frequency but vary independently, causing the phase difference between them to wander over time. Phase coherence is high when both the mean rate of phase increase and its variations are common between sites, and when phase slips are corrected so that sites maintain a fixed phase difference (in particular, zero phase difference). We verified that high phase coherence was not attributable to recording devices at one location “overhearing” cicadas at another location (Appendix S4).

Second, to find the characteristic distance scale over which cicadas were synchronized at time *t*, we compared the matrix of phase agreement values $\text{Re}\left(e^{i\left(\phi_{j,t}-\phi_{k,t}\right)}\right)$ between locations indexed by *j* and *k*, with a matrix $e^{-\delta_{\mathrm{jk}}/d}$ , where $\delta_{\mathrm{jk}}$ is the distance between the locations. This represents an exponentially falling agreement profile; that is to say, that phase agreement falls exponentially with distance between a pair of sites, with some distance constant *d* such that the phase agreement is expected to be equal to 1 at distance 0, and 1/*e* at distance *d*. Then, we determined the value of d at each moment in time that best matched the observed phase agreement values. For each time, *t*, we found the value of *d* for which the Pearson correlation between the observed phase agreement matrix and the profile matrix was maximized. This was considered the characteristic distance scale, *d*(*t*), of synchrony. However, maximal *R* ^2^ was low (average 0.0967 over time); *d* reflects a forest‐wide characteristic distance from which individual pairs of locations could deviate.

Third, spatial synchrony was patchy, with patches internally phase‐coherent but having different phases from each other. For this reason, we developed an entropy‐type measure reflecting the tendency to “patchiness” of phase values at any point in time. Our measure is analogous to nearest neighbor (Kozachenko, 1987), and *k*th nearest neighbor (Singh, Mistra, Hnizdo, Fedorowicz, & Demchuk, 2003) approaches to the Shannon entropy. Details are provided in Appendix S5.

Significances of the Pearson correlations between the brightness profile and several cicada activity time series were evaluated by generating amplitude‐adjusted Fourier transform (AAFT) surrogates (Schreiber & Schmitz, 2000) of the brightness profile. For each surrogate, 5‐min moving averages were computed, parallel to what was done to generate the brightness profile itself. Correlation with the actual brightness profile was then compared to the distribution of correlations with surrogates. This is a standard approach to account for autocorrelation (Schreiber & Schmitz, 2000).

### 2.3 Models

We supposed that the cicadas could be modeled as relaxation oscillators, alternating between periods of not calling and periods of calling. The duration of not calling depends on interactions between the cicadas; specifically, each cicada is sensitive to the sound volume of the other cicadas. The average rate and the sensitivity to sound volume were variable parameters. In this simulation, we demonstrated how a tendency of individuals to call sooner when sound volume is high resulted in the synchronization of cicadas within a tree (cycling behavior) and how hearing neighboring trees caused the trees to come into phase (synchronous behavior). Increasing the overall calling rate (i.e., due to a widespread stimulus such as change in illumination) increased synchrony. See Appendix S6 for more details.

To investigate the effect of more severe sound attenuation in the forest, we ran alternate simulation 1 with the cicadas in each tree only able to hear nearest neighbor trees in the grid. To investigate the effect of delayed signal propagation due to the finite speed of sound, we ran alternate simulation 2 with the cicadas able to hear only the past state of each tree, with the delays set to simulate a forest 300 m across (75‐m separation between trees), matched to our greatest interlocation distance on the main site. Finally, in alternate simulation 3, we increased the delay by a factor of ten (i.e., the time delay associated with signals coming from the far ends of the forest was considerably greater than the calling period), to verify that a large delay prevented the establishment of synchrony.

## 3 RESULTS

The cycling of cicada chorus volume varied in frequency over time. Wavelet transforms of cicada sound volume at our recording locations all showed a clear feature (the bright local maximum in the magnitude of the plotted quantity at each point in time, forming a ridge running through the surface plot) at around 0.15–0.2 cycles per second, equivalent to a peak in volume every five seconds or so (Figures S2–S21, note the similar temporal variation in cycling frequency between locations). The activity at individual sites was intermittent, so a well‐defined cycling frequency cannot always be traced through time in the wavelet transform of a single site. However, plotting the average (over locations) of the squared amplitudes of all the wavelet transforms clearly shows a single peak with varying frequency (Figure 1). We traced this peak through time, the “forest frequency,” and took the amplitude of the cycles in sound volume (distinct from the volume itself) and the phase of these cycles from each wavelet transform at each point in time at the forest frequency. Having a magnitude and a phase, these are complex‐valued functions of time which can be regarded as analogous to a wavelet component, but extracted at a frequency which varied through time (according to how frequencies of cicada volume fluctuations varied) instead of at a fixed frequency. See Supplementary information for a video of the evolution of the relative phase values over the whole period of measurement (see also Figure S22 for key).

**FIGURE 2:**
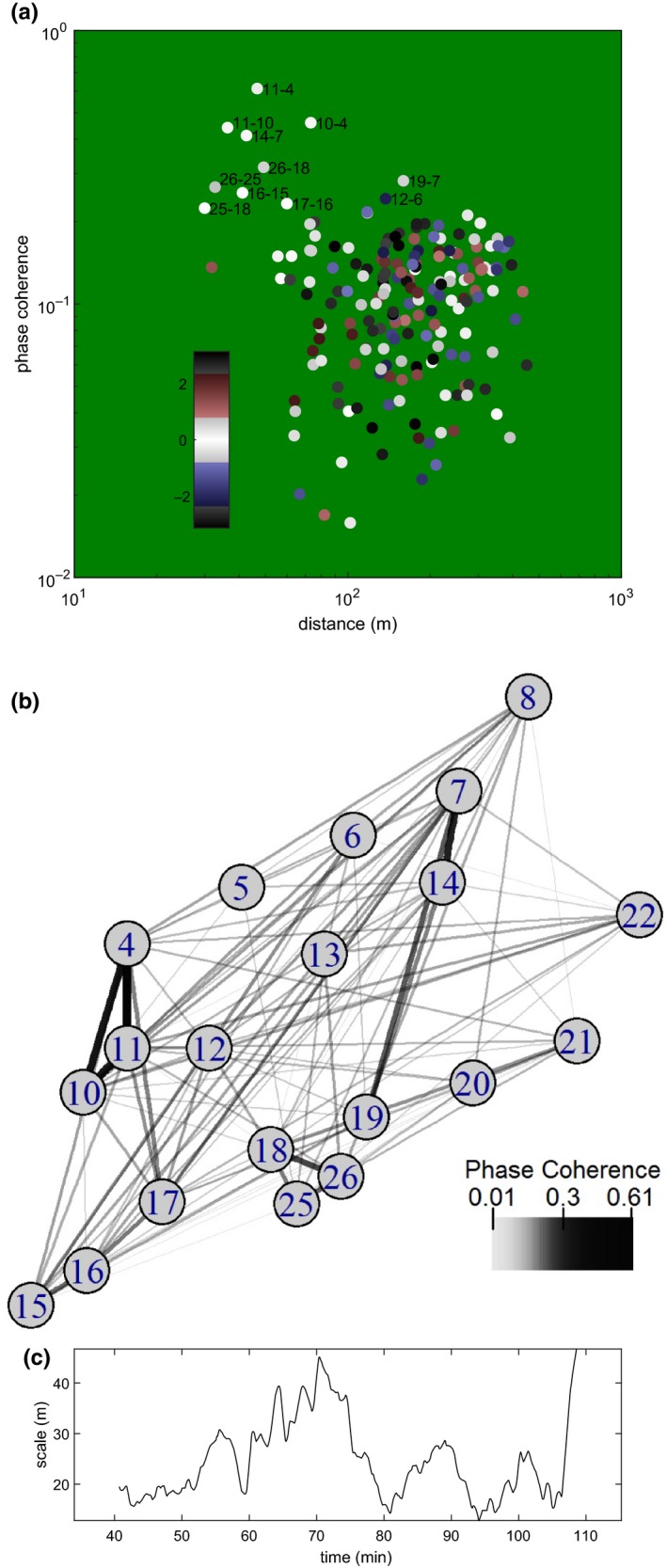
Spatial synchrony falls with increasing distance. (a) Circles show phase coherence of pairs of transforms drawn from different locations, plotted against physical distance in space. Color indicates average relative phase, in radians. Note that high‐phase coherence pairs are in phase (white). The top ten highest‐phase coherence pairs are labeled with location numbers. (b) Strength of phase coherence between points on the map. (c) Characteristic distance scale of phase agreement in the forest as it varies in time (see Methods: Statistics)

Having obtained the wavelet component analogues of the previous paragraph, we evaluated the synchrony, over all time, of all pairs of such components from different locations, using the phase coherence technique, and we verified synchrony was not due to a location “overhearing” cycling at a nearby location (Methods: Statistics; Figures S22 and S23). High values of phase coherence were obtained for several pairs of locations in close proximity, whereas more distant pairs of locations typically had low‐phase coherence (Figure 2a,b). Note the logarithmic scale of the coherence axis in Figure 2a: most sites separated by long distances manifest low coherence values with an arbitrary typical phase difference. These pairs of sites provide a baseline of asynchronous examples against which the high values of coherence (which we observe to exhibit typical phase difference zero) for neighboring sites can be judged. The neighboring sites were able to maintain or recover typical phase differences near zero, at the forest frequency, despite the intermittency of local cycling. The spatial extent of synchrony varied through time (Methods: Statistics; Figure 2c), with values in the 10 s of meters. The most coherent pair of sites was 4 and 11, 47 m apart.

**FIGURE 3:**
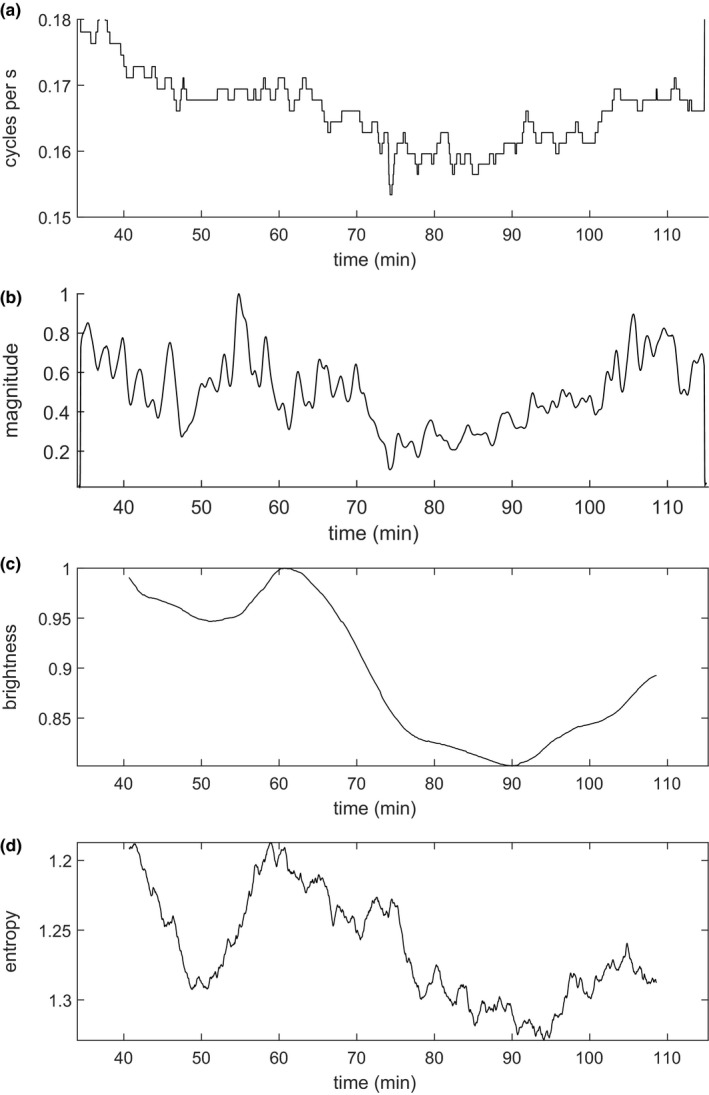
Cicada variability and its covariates. (a) Average cicada volume cycling frequency (Hz). (b) Normalized average amplitude of volume cycling. (c) Normalized, smoothed (moving‐average) brightness. (d) Smoothed (moving‐average) entropy of the distribution across locations of the phases of volume cycling (normalized units)

Fluctuations in ambient light appeared to drive temporal variation in cicada volume cycling frequency and cycle phase synchrony. The temporal variation in cycling frequency and cycling amplitude extracted from the average of the wavelet transforms over all the locations are shown in Figure 3a,b, respectively. Light level, taken from image brightness of a video recording of the forest canopy (location 5 on Figure 2b, see Methods: Data), is shown in Figure 3c. We found a significant correlation between light level (Figure 3c) and cicada cycling frequency (Figure 3a; *p* = .0055) and amplitude (Figure 3b; *p* = .0457), using a Fourier randomization‐based test that properly accounts for temporal autocorrelation in these quantities (Methods: Statistics).

We also computed a measure of the entropy, that is, the level of randomness or spread or nonalignment, of the phases of cicada volume oscillations at recording locations; the measure was a function of time and smoothed using a 10‐minute moving average (Methods: Statistics; Figure 3d and Figure S24). At any moment in time, cicada cycle phase values demonstrate local relationships but do not agree on any global value. Entropy is maximal when they are completely evenly distributed and is smaller when local phase similarities are greater. The entropy measure was negatively correlated with brightness (*p* = .0059, again accounting for temporal autocorrelation of these quantities; note the inversion of the vertical axis in Figure 3d). High levels of illumination were apparently associated with faster, larger magnitude, and more spatially synchronous cicada chorus volume fluctuations. The spatial extent of the influence of illumination fluctuations was at least 3 km, since a simultaneous off‐site recording almost 3 km away (Methods: Data) showed changes in the frequency of cicada volume fluctuations that matched those of the 20 main recordings (Figure S25). The characteristic distance scale over which phase relationships between locations fall off (Figure 2c) was not significantly correlated with brightness (*p* = .2230), possibly because of uncertainty in the distance‐scale estimate (Methods: Statistics). This measure depended on phase coherence between all pairs of sites, but only some pairs showed high‐phase coherence. For instance, locations 10 and 4 had high‐phase coherence but were relatively far apart (73 m).

**FIGURE 4:**
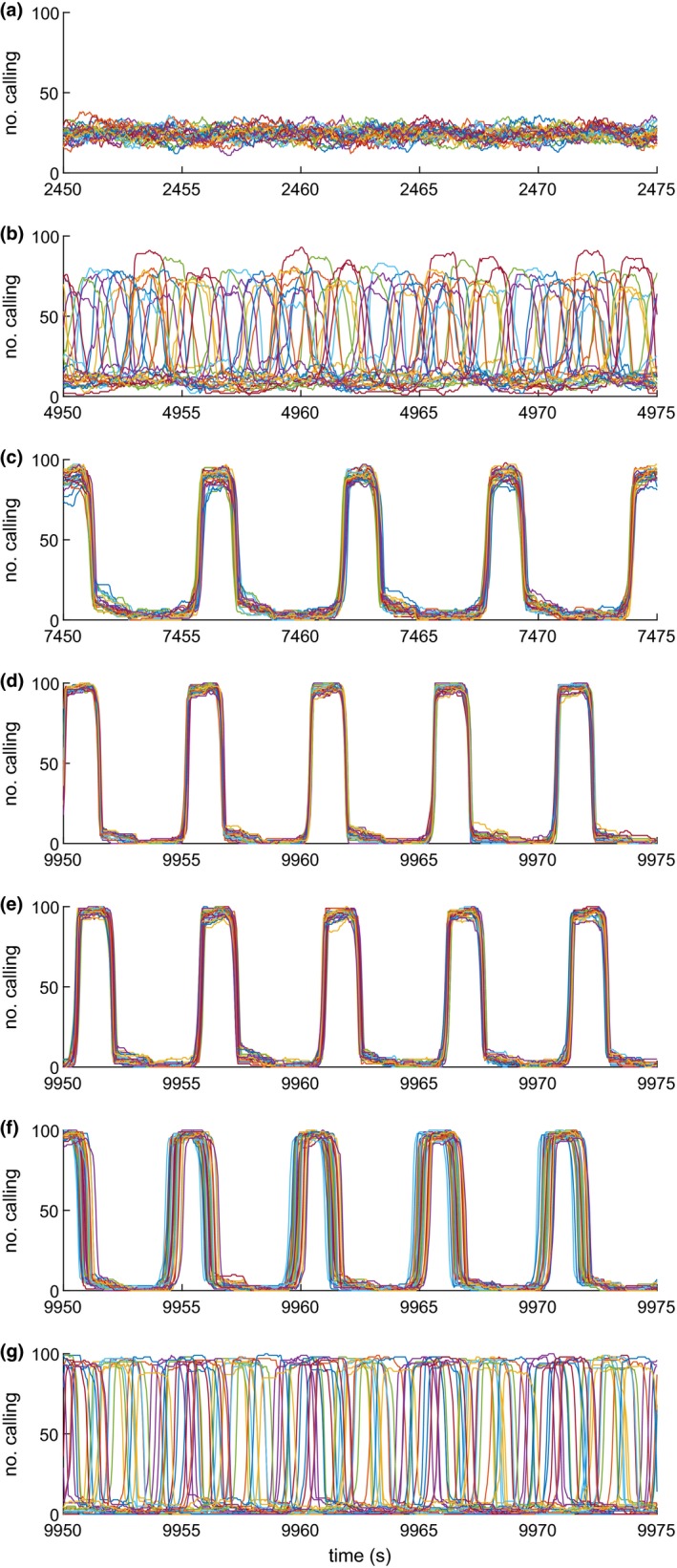
Segments of data from example simulations showing temporal variation in number of cicadas calling in each of 25 trees. (a) Cicadas cannot hear each other's calls, resulting in no synchrony. (b) Cicadas can hear calls from their own tree, but not calls from other trees, resulting in tree‐level synchrony. (c) Cicadas can hear calls from their own tree and other trees, resulting in forest‐level synchrony. (d) Cicadas can hear calls from their own tree and other trees, and individual rate of calling is increased, resulting in faster and larger volume cycles and increased synchrony. (e) Final segment of alternate simulation 1: cicadas can hear only from their own tree and adjacent trees, still resulting in forest‐level synchrony. (f) Final segment of alternate simulation 2: cicadas hear from other trees with a sound delay consistent with our test site, still resulting in forest‐level synchrony. (g) Final segment of alternate simulation 3: cicadas hear from other trees with a ten times greater sound delay, resulting in no synchrony

An interacting relaxation‐oscillator model (Methods: Models) simulating a forest patch with 25 regularly spaced trees each occupied by 100 cicadas was used to explore possible mechanisms of the observed synchrony and changes therein with illumination. Each cicada calls for 1.5 s and then builds up to call again at its own rate, subject to some random variability. Representative data segments are taken from a model in which various effects are switched on one by one (Figure 4a‐d). In this way, in each segment the system is allowed to reach an equilibrium absent a given model feature, before that feature is introduced in the next segment. If a cicada's threshold for calling is reached earlier according to how many other cicadas are calling in the same tree, calls become synchronous in each tree (Figure 4b). If cicadas are also influenced by the sound of all the other trees, the cycles in the trees become synchronous (Figure 4c). Finally, externally decreasing the mean characteristic period between calls from 5 to 4 s (increasing intrinsic calling rate) produces an increase in total calling activity, faster volume cycling, even more pronounced cycles, and more spatial synchrony between trees (Figure 4d). These effects occur together, consistent with our empirical data (Figure 3). Separately, we checked a range of model characteristic periods (calling time plus time between calls) from 5 to 7 s, obtaining consistent results. It appears that all the behavioral covariation seen in Figure 3 can be explained by a single behavioral mechanism: an increase in the calling rate of individual cicadas under stronger illumination.

The model represented in Figure 4a‐d includes the attenuation of sound signal strength as it propagates through the forest, in the form of both the inverse square law and an exponential decay representing sound absorption by the physical environment. The decay coefficient and thus the strength of the influence of neighboring trees on the cicadas in each tree were chosen plausibly but somewhat arbitrarily (Methods: Models). The result was complete synchrony, unlike the local and episodic synchrony of our empirical measurements. To investigate whether the model was biased by permitting too much acoustic influence between trees, we ran alternate simulation 1, permitting cicadas to hear only the sound from nearest neighbor trees. The end state (Figure 4e) was still comparable with Figure 4d: complete and stable synchrony. For context, note that work on small‐world networks in theoretical population dynamics models indicates that nearest‐neighbor‐only connections can allow more efficient global synchronization than permitting more extensive connection networks (Ranta, Fowler, & Kaitala, 2008).

Another possible barrier to the establishment of synchrony is the time delay associated with the speed of sound, not included in simulations above. In alternate simulation 2, we set the grid of trees in the model to be 300 × 300 m (comparable to the scale of our observations), imposing over a second of delay on the sound transmitted between opposite corners of the forest. But, this modification was not sufficient to prevent synchrony (Figure 4f). Finally, in alternate simulation 3, increasing the delay by ten times does prevent a stable synchronized state (Figure 4g). Cicadas in Clinton Park showed highly variable activity levels over the recording period, and there may not have been time during each epoch of synchronization for spatial synchrony to reach the maximal extent permitted by the limitations of sound travel time. Further reflection on the reasons for local, as opposed to global synchrony in our empirical data, is in the Discussion.

## 4 DISCUSSION

We showed how *M. cassini* self‐organizes into cycling choruses which respond collectively to illumination. Simple dynamical principles result in several changes to chorusing and its synchrony. Specifically, our results provide good evidence for the hypothesis that a simple change in calling rate, responsive to illumination levels (a type 1 environmental synchronization), alters the strength of aural stimulus to produce changes in the amplitude and frequency of volume fluctuations and in their tendency to synchronize spatially (a type 2 synchronization). The correlated change in calling rate is not in itself sufficient to yield type 2 spatial synchrony, as two locations can in principle have the same mean calling rate but maintain no particular set phase difference between their cycles. However, as demonstrated in our models, the responsiveness to sound stimulus of the individual cicadas which yields local spatial synchrony also yields increased type 2 spatial synchrony when the collective calling rate is higher. The significant correlations between observed light levels and several measures of cicada activity provide evidence that cicadas are responding to illumination rather than air temperature or another correlated cue, as the response is rapid and consistent. This investigation was limited to a single brightness‐recording device, at location 5. This device had a wide‐angle view of the canopy and through the canopy to the cloud layer over the forest. Brightness fluctuations were smoothed over time to eliminate local fluctuations due to movement of leaves, etc. We note that the average cycling rate of the cicadas throughout the forest was correlated with this single brightness recording, helping confirm the assumption that the cicadas throughout the area of measurement were subject to the same variation in brightness. But additional spatially distributed devices could be deployed in future work to investigate the possibility that perceived sky brightness may vary spatially as well as temporally.

*M. cassini* provides an excellent simultaneous example of three very different types of synchrony: 17‐year self‐organizing type 2 phase synchronization of emergence on large spatial scales; 5‐second type 2 self‐organizing phase synchronization of volume fluctuations on small spatial scales; and medium‐scale (at least 3 km) type 1 synchrony of changes in the nature of the volume oscillations, due to changes in illumination. The latter two types are newly revealed here. Synchronous calling and volume fluctuations of *M. cassini* are apparent to casual observers. The spatial extent of synchrony was unknown, as it is not possible for a single observer to determine whether cycles at two locations are phase‐synchronized. The use of 20 recording devices, facilitated by a citizen‐science approach and smartphone technology, demonstrates the extent of self‐organized phase synchronization, and the even greater extent of synchronous changes in cycling amplitude and frequency.

The calling behavior of male cicadas is an information transmission phenomenon, an important but understudied aspect of ecology (O'Connor et al., 2019). The phenomenon of volume cycling and its spatial synchrony ultimately results from the responsive calling behavior of individual insects, and the evolved chorusing behavior of male cicadas is interdependent with mate selection of the females. From a behavioral point of view, male cicadas must integrate information about the calling of other cicadas into their own behavior to synchronize. Female cicadas then incorporate information about male calling patterns into their mating behavior. From an evolutionary point of view, information about the mating preferences of females is incorporated into the information processing behavior of males in the next generation by the process of natural selection. Several possible selection strategies may render the tendency to call when other males are calling (resulting in synchrony) adaptive. One possibility is that females are unable to distinguish individual cicada voices in the audio field, and simply fly to the tree with maximal oscillation and/or maximum peak volume. Males thus cooperate to draw females to their tree or area, outcompeting out‐of‐synchrony individuals. Only *M. cassini* cycles, and the females of this species may have to identify it amid calling cicadas of two other *Magicicada* species. Possibly, large volume oscillations (maximal peak‐to‐trough differences in sound volume over the course of a chorus cycle), as opposed to simply high average sound volume, enable species discrimination. Another possibility is that females actively discriminate in favor of males with an ability to synchronize with the chorus, as an indicator of fitness. Work on other insect taxa has raised the possibility that synchronous calling can arise as an indirect result of individuals attempting to demonstrate individual fitness by calling “first” (Snedden & Greenfield, 1998), but this work was not on cicadas. Further detailed research would be required to determine which of these mechanisms is accurate, if any. In the case of our relaxation‐oscillator model, we have assumed that sound volume is a stimulus to calling, without assuming the evolutionary basis for this response. But we believe that our observations and modeling constitute a first step, as described in the Introduction, for a research program in which mechanisms of synchronous chorusing are tested by comparing predictions of spatiotemporal synchrony patterns that should be generated by these mechanisms to the actual patterns we observed.

Although our simulations parallel our observations in important respects, they differ in other respects. Unlike our simulations, observed *M. cassini* volume cycles are episodic and only locally synchronous, rapidly fading and reestablishing and synchronous only to a spatial extent of tens of meters. These characteristics are probably intrinsic to the choruses, but variable in their details according to specific local conditions. The most coherent site pairings identified in Figure 2a belong to spatially localized clusters. Most notable are the 11–4, 11–10, 10–4 cluster, the 14–7 pair, the 26–18, 26–25, 25–18 cluster, and the 16–15, 17–16 cluster. The particular coherence values obtained between each pairing are time averages and may depend on the overall level of *M. cassini* activity at each location, and its intermittency, as well as the strength of coupling with neighboring sites due to proximity. Site 19 appears to maintain high coherence with site 7 (and 14) despite distance and the existence of a road running through the forest between site 19 and the 7–14 pair. This may be because the highly synchronous site 7, 14 cicadas are part of a loud and influential chorus, audible to the cicadas at site 19 across the open space of the road. It appears that spatial proximity alone does not determine the strength of spatial synchrony, though it does determine much of it. We also observed that noise disturbance (a helicopter flyover) had a depressive effect on chorusing.

The finite speed of sound and the sound‐absorbing/sound‐attenuating effects of trees should tend to render synchrony local in a real forest. If sound did not attenuate, and individual insects were to wait and call as the sound from distant cicadas arrived, this would result in an inevitable breakdown in simultaneity: sound from different distances arrives with different delay times. Exponential sound attenuation is sufficient to ensure that the majority of sound energy reaching a given cicada originates within a finite radius, making local synchrony possible. Even at the largest distance scales examined here, our simulations indicate that sound travel delay time (less than half the period of a cicada cycle over our distances) is not likely to be a limiting factor. We note that increasing the number of trees in our model tended to increase the time required for the forest to reach a stable synchronous state. We hypothesize that our cicadas are capable of greater synchronization but did not fully synchronize in our data due to the intermittency of their activity on the day of measurement. Our recordings were performed on an overcast day with variable illumination toward the end of the mating season. Further work should investigate the adaptation of synchronous chorusing behavior to the local soundscape and landscape, and should measure cicada activity under brighter illumination and earlier in the season, when we predict spatial synchrony to be even clearer and more spatially extensive than we observed.

## CONFLICT OF INTEREST

The authors declare no conflict of interest.

## AUTHOR CONTRIBUTIONS

**Lawrence** **W. Sheppard:** Conceptualization (equal); data curation (lead); formal analysis (lead); investigation (equal); methodology (equal); software (lead); visualization (lead); writing—original draft (lead); and writing—review and editing (lead). **Brandon Mechtley:** Conceptualization (equal); data curation (supporting); investigation (equal); methodology (equal); software (supporting); visualization (supporting); and writing—review and editing (supporting). **Jonathan Walter:** Investigation (supporting); methodology (supporting); software (supporting); visualization (supporting); and writing—review and editing (supporting). **Daniel C Reuman:** Conceptualization (equal); formal analysis (supporting); funding acquisition (lead); investigation (equal); methodology (equal); project administration (equal); resources (equal); supervision (lead); and writing—review and editing (equal).

## Supporting information

Supplementary Material

Video S1

Video S2

## Data Availability

The supporting data (cicada sound volume time series as used in the primary analysis) have been placed in the accessible online repository Dryad: DOI, https://doi.org/10.5061/dryad.4f4qrfj86.
