# Supplementary material for: Self‐organizing cicada choruses respond to the local sound and light environment
Source: Ecol Evol. 2020 Apr 20;10(10):4471–82. doi: 10.1002/ece3.6213 (PMC7246199; doi:10.1002/ece3.6213)
Supplement: Supplementary file 1 — Supplementary Material [file ECE3-10-4471-s001.pdf]

Supplementary Information

Lawrence W Sheppard[a], **Brandon Mechtley**[b], **Jonathan A Walter**[c], **and Daniel C Reuman**[a,d]

[a]University of Kansas Department of Ecology and Evolutionary Biology and Kansas Biological Survey; [b]Arizona State University School of Arts, Media and Engineering; [c]University of Virginia Department of Environmental Sciences; [d]Rockefeller University Laboratory of Populations

This manuscript was compiled on March 3, 2020

cicada | synchrony | wavelet | insect chorus | citizen science

1. S1. Additional information on cicada outbreaks and calling

The 17 year emergence period is an extreme example of synchronized progression through life stages. Although absolute numbers of cicada young under the ground inevitably decline over the years after the last emergence, the emergence phenomenon itself is ecologically comparable to an outbreak year in a population subject to periodic variability. *Magicicada spp.* are unusual in that each 'outbreak' corresponds to a single generation of cicadas, distinct from other periodically outbreaking species such as pests in agricultural systems (Cooke *et al.*, 2007; Singh & Satyanarayana, 2009). The periodicity of many pest species is irregular and subject to factors such as forest type (Johnson *et al.*, 2006), climate (Esper *et al.*, 2007) and feedbacks with the plant community (Kamata, 1998). Unlike outbreaking of annual herbivorous insects, emerging cicadas have effectively stockpiled resources below ground over their 17 years of growth, before releasing them in a pulse above ground while producing the next generation of cicada eggs. The overwhelming numbers of cicadas active during emergence years may itself produce spatially synchronous effects on vegetation. Koenig & Liebhold (2003) report systematic depression of tree growth in outbreak years, although the effect is small (Shelton & Winkle, 2009). Cicada emergences can have complex effects on predators such as birds (Koenig *et al.*, 2011), and Storm & Whitaker Jr. (2007) report that cicadas become a major food resource for mammalian predators during outbreak years. Several explanations exist for the extraordinary synchronized periodic emergence (Lloyd & Dybas, 1966), such as hybridization avoidance (Cox & Carlton, 1988), predation avoidance (Hoppensteadt & Keller, 1976; Goles *et al.*, 2001; Koenig & Liebhold, 2013), and avoiding the build up of pathogens which can persist between years (Duke *et al.*, 2002).

Aggregation of males followed by mate selection by females is a common phenomenon (called 'lekking' (Walker, 1983), such as by Black Grouse (Fiske *et al.*, 1998; Alatalo *et al.*, 1991)). However, there is debate as to whether insect aggregations constitute lekking (Höglund & Alato, 1995). Cicadas make sound by a variety of mechanisms (Myers, 1929; Pringle, 1953; Young, 1983; Luo *et al.*, 2015), and *M. cassini* in particular is highly adapted to sound generation (Nahirney *et al.*, 2006). Sound production in animals is limited by physiological and energetic constraints (Gillooly & Ophir, 2010), with corresponding consequences for their behaviour. Temperature variation can affect cicada sound production in particular: sound power is observed to correlate with body temperature (Sueur & Sanborn, 2003) in *Tibicina* species and syllable rates depend on temperature in the genera *Tettgetta* and *Tympanistalna* (Fonseca & Allen Revez, 2002). High performance sound generation in *M. cassini* is achieved by means of a superfast striated muscle acting on a dedicated tymbal organ in the first abdominal segment (Nahirney *et al.*, 2006). Communication patterns within a chorus may be complex (Oberdorster & Grant, 2007). Acoustic identification is a useful and reliable method in the field (a library of European cicada calls has been made available (Gogala, 2006)) and has even been used to uncover new species of cicadas (Alexander & Moore, 1962). The recent increase in the use of smartphones has made possible semi-automated crowdsourced identification programs (Zilli *et al.*, 2014).

2. S2. Sound data acquisition and processing

We began with 26 devices, mostly smartphones, optimised for voice recording using a variety of recording software with a typical sampling rate of 44100 Hz. Sound waveforms were either recorded as .wav files or converted to .wav prior to further analysis. While approaching the main recording site, we activated two recording devices ahead of time and placed one in a woodland patch discontiguous from the main recording site, as a long baseline reference. This off-site recording was made at a distance of 2960m east of location 8 (GPS coordinates 38.94244, -95.34922), and its transform is presented in Fig. S25 for comparison with the other recording locations.

To ensure our ability to compare the cycle phase of cicadas at two widely separate locations with exact timing, we activated the devices and ensured that both recorded a single preliminary reference event (a balloon pop) and recorded continuously thereafter. Recording devices were scattered evenly along forest paths and placed at ground level, then left undisturbed for the duration of measurement.

The recording of a secondary reference event on the devices after they had been collected again enabled us to verify later that all devices had recorded the same length of time between reference events and that their timings were thus comparable. One older device was rejected at this stage for 'losing time'. Four other devices were rejected for losing data due to equipment failure, logistical problems, or human error. Twenty useable locations were identified for further analysis.

We evaluated the sound spectral profile of the cicadas at each location we recorded, and applied adaptive non-causal filters to isolate the sound content associated with the cicadas, as follows. Audio data was divided into 0.1 second segments and

DRAFT

the sound power spectrum was found within each. We trimmed off data from outside the period when all recording devices were active and undisturbed. Each segment was Fourier transformed to identify the sound frequencies present. The total power inside a band (2 to 7 kHz) that matched the cicada spectrum was evaluated for each segment (this is called 'band-pass filtering'). We found the mean sound power spectrum (by Fast Fourier Transform) of all segments, and took the associated amplitude of each frequency in the range associated with the cicadas to make a filter profile. Mean sound power spectra are shown in fig.S1. Next, for every segment we multiplied the Fourier transform coefficients at these frequencies by this profile, prior to calculating its sound power. This multiplication exaggerates the contribution due to the cicadas at the expense of other intermittent sounds (this is called 'matched filtering'). Investigating cicada sound production, Nahirney *et al.* (2006) reported the characteristic frequency to be 7.1-7.4 kHz, higher than the spectral peak found in our cicada recordings. The resonant frequency of the resonant cavity (which amplifies the sound) may not match the tymbal frequency (generated sound), which might explain why the actual sound output of our cicadas does not precisely match these previously reported values.

The cicada sound volume values of the segments formed a time series which we then wavelet-transformed. Each time series of cicada sound volume was de-meaned and normalized to have unit variance, then wavelet transformed with a complex mother wavelet having a center frequency of $f_0 = 4$ (Addison, 2002). Individual transforms of data before trimming, after trimming, after 2 to 7 kHz band-pass filtering, and after matched filtering are included for all recording locations at the end of this document, showing the effect of the cleaning process on the wavelet transform in each case (Figs S2-S21 and S25). A diagram showing the steps in data cleaning is presented in Fig. S26, and the wavelet transform results (shown in Figs S2-S21d) are presented in one combined figure in Fig. S27.

Relative amplitude and phase values of wavelet transforms for a particular point in time are plotted for each location in figure S22, and the evolution of these values over time is shown in forestclip.mp4. The mean sound power spectra of the locations are compared in figure S1. The mean wavelet energy plot over all locations was then obtained, and the average cicada cycling frequency in the forest was found at each point in time (the maximum in this plot for each time). For each location, at each point in time, we extracted a wavelet value giving the magnitude and phase at this variable frequency.

3. S3. Canopy light level

The sky was overcast and varied slowly in brightness over time, although the recording contains several sharp discontinuities associated with the video function automatically adjusting exposure. We took the mean pixel brightness of the recorded image as an indicator of sky brightness for the whole forest. Discontinuities were identified and removed by hand, with the segments on either side moved back into alignment. This produced a smoothly varying light curve, with some variability due to wind and other canopy disturbance. Although the field of view was partly obstructed by the canopy overhead at this observation location, the area of sky seen through the gaps in the canopy corresponds to a wide field of view - a patch of cloudy sky sufficient to cover the ground area of our observations. This approach was vindicated by the mean cicada activity being found to correlate with the sky brightness measured at this central location.

We tested the correlation coefficient between the light curve and the changing average cycling period of the whole forest. To evaluate statistical significance we generated artificial 'surrogate' light curves by rearranging the values from the light curve in random ways, so as to approximately preserve the spectral characteristics and autocorrelation function of the light curve, using an amplitude adjusted Fourier transform method (Schreiber & Schmitz, 2000), and then repeating the moving average (Methods) to get a curve of comparable autocorrelation. We also tested for a correlation with average amplitude of cycling in the same way.

DRAFT

4. S4. Testing for 'overhearing'

To check that observed phase coherence was due to spatial synchrony between the cicadas in the neighborhood of each recording location, and was not attributable to microphones 'overhearing' cicadas from neighboring locations, we checked the most phase-coherent locations, 4 and 11, and examined temporal variability of cycling amplitude at each location along with temporal variability in phase agreement between these locations. We selected these sites as being close, with high apparent synchrony of sound cycling. As usual, we are limited by the use of cellphone recorder hardware and software that does not give us an absolute measure of sound energy, but can be used to follow relative changes, sufficient to track the 5 second cycling and changes in the amplitude of such cycles.

In this check we suppose that most of the sound energy being recorded at each site originates 'locally', ie. in the immediate vicinity of the microphone, and some small fraction (perhaps zero) originates at the site ostensibly monitored by the other microphone. Both sites are found to vary over time in terms of the volume of sound and the amplitude of cycle oscillations which are recorded, and this variation is at least partially independent, indicating that at least some of the sound and the cycles originate locally at each site.

When two independently varying (unsynchronised) signals are combined in this way, the correlation between the signal mixtures is maximised when the amplitude of the signal originating at one location is maximised and the other is minimised. This does not occur if the signals are not independent (synchronised).

Thus in the case that apparent synchrony is due to microphone crosstalk with independent local sound sources, we would

expect the cross talk to produce maximum apparent synchrony when the independent local cycles are 'quiet' and the distant source 'loud', with the local sound being 'drowned out' by the sound from the alternate source. The limiting case would be local silence, and the resultant (relatively quiet) recording being 100 percent due to sound from the distant source, giving 100 percent synchrony.
This effect is not observed at either site. When either of the local cycling powers drops below average, the synchrony level drops below the level found when both are above average. This is in accordance with our simulations in which high amplitude cycling more easily demonstrates (true) synchronisation.If phase coherence was attributable to 'overhearing', we would expect phase agreement to be strongest when cicadas at one location were producing large-amplitude volume fluctuations and those at the other location were not, resulting in recorded cycling in the second location being more dominated by the strong fluctuations at the first location. Defining phase agreement at time t as $\mathrm{Re}(e^{i(\phi_{4,t}-\phi_{11,t})})$, where $\phi_{4,t}$ is the phase of volume oscillations at location 4 and $\phi_{11,t}$ is the phase at 11, we divided each set of transform values into the half for which the amplitude was above average and the half for which it was below average. We checked the phase agreement values obtained when: location 4 had above average cycling amplitude and location 11 had above average cycling amplitude (Fig. S23a); location 4 had above average cycling amplitude and location 11 had below average cycling amplitude (Fig. S23b); location 4 had below average cycling amplitude and location 11 had above average cycling amplitude (Fig. S23c); and location 4 had below average cycling amplitude and location 11 had below average cycling amplitude (Fig. S23d). The highest agreement was found for Fig. S23a, indicating that synchrony was greatest when cicada volume cycling at both locations was high. Agreement was lower for Fig. S23b, indicating that the observed agreement was not due to the cicada sound from location 4 being recorded at location 11. Similarly, agreement was lower for Fig. S23c than for Fig. S23a. The lowest agreement was for Fig. S23d.

5. S5. An entropy-based measure of phase clumping

We applied an entropy-based measure of non-uniformity in the whole-forest phase distribution. It is based on the approach of Kraskov *et al.* (2004) to calculating the entropy of a distribution (of, in our case, phase values) by ordering N observed values and working with their first differences. The first differences between the sorted phase values sum to 1 turn around the circle and are small inside clusters of similar phases, large outside. The entropy of the distribution of phases at a moment in time in the forest is

$$H(f) = -\int_{\phi} f(\phi)\log(f(\phi))d\phi. \quad [1]$$

Approximating the probability density $f(\phi)$ between (sorted) observed values ϕ_n and ϕ_{n+1} as $\frac{1}{N(\phi_{n+1}-\phi_n)}$ (equivalent to assuming a 1 in N probability of a value falling between ϕ_n and ϕ_{n+1}) we can approximate entropy as

$$H_{\mathrm{estimate}}(f) = -\sum_{n=1,...,N} \frac{(\phi_{n+1}-\phi_n)}{N(\phi_{n+1}-\phi_n)} \log\left(\frac{1}{N(\phi_{n+1}-\phi_n)}\right) \quad [2]$$

$$= \frac{1}{N}\sum_{n=1,...,N} \log(N(\phi_{n+1}-\phi_n)). \quad [3]$$

Here the summation is over $n = 1,\ldots,N$ and ϕ_{N+1} is interpreted as referring to ϕ_1, given the circular nature of phases. Phase differences are computed modulo 2π. In principle, this approach could fail as phase values of locations grow at different rates over time and sometimes overtake each other, implying infinite probability density when two values become equal. In practice, none of our phase values are exactly equal but the entropy estimate is 'spiky', including many transient peaks (Fig. S24). Another approach would be to base the entropy estimate on the spacing between k^{th} nearest neighbors (Singh *et al.*, 2003), but this is inappropriate for our weakly synchronous system where we see only pairs and small clusters of locations with high synchrony. Evaluating entropy based on the kth nearest neighbor would miss clusters of size less than k. We calculated the entropy estimate for each 0.1 seconds using the phase values drawn from the wavelet transforms for each 0.1 seconds, and then we smoothed by a moving average over 10 minutes.

6. S6. Spatially synchronous models

Our model cicadas were encoded by variables representing how far through the calling or not-calling period each cicada had reached at a point in time. When calling, each cicada would count down at a fixed rate until calling ceased, with a total calling time of 1.5 seconds. When not calling, each cicada would count up, at a variable rate, to a total that could depend on the volume of sound produced by other cicadas at that moment in time; then it would recommence calling. We modeled 25 trees, each with 100 cicadas. The cicadas were simulated over 10,000 seconds with a time step of t_{step}=0.05 seconds. The first state variable of cicada m, $\xi_m(t)$, was non-zero only if the cicada was calling, and counted down from 1 to 0 over 1.5 seconds until the cicada stopped calling. The second variable, $\psi_m(t)$, was a phase variable that accumulated from 0 to 1 when the cicada was not calling. This variable increased by a variable increment each time step. The increment was equal to a constant c plus the sum of a randomly determined fixed increment which was unique to each cicada and a randomly determined temporally varying increment:

$$\psi_m(t+0.01) = \psi_m(t) + c + 0.0002\epsilon_m + 0.0075\upsilon_{m,t}. \quad [4]$$

DRAFT

Here $c = 0.01$ and ϵ and υ were both normally distributed random numbers with mean zero and variance 1. One value of ϵ_m was produced, once and for all, for each cicada; this was done independently for different cicadas. The $\upsilon_{m,t}$ were independently produced for different values of m or t. When ψ_m reached a threshold of 1 full cycle, $\psi_m = 1$, it was reset to zero while the cicada called for 1.5 seconds. Thus an average cicada will call for 1.5 seconds exactly then rest for approximately 5 seconds, then repeat. Variability (and asynchrony) in calling rate between cicadas results from variable resting time.

With no interactions between the cicadas, we used a burn-in to reach a stable distribution of calling/not calling cicadas to use as a start point. For the first part of the simulation (0 to 2500 seconds), the cicadas called randomly.

We then introduced a series of interactions, which caused cicadas to call, and ψ to reset to zero, prior to reaching one full cycle. The sound of other cicadas was taken to reduce the calling threshold by an amount proportional to sound volume; cicadas could now hear other cicada calls, encouraging them to call too. In each tree, the sound volume level, V_i, was set equal to the fraction of cicadas that were calling in that tree, v_i. This sound stimulus reduced the threshold (previously at $\psi = 1$) for a cicada in that tree to resume calling, by $0.2V_i$. This change caused the cicadas in a tree to become synchronous. Thus the second quarter of the simulation (2500 to 5000 seconds) was dominated by periodic cycling of cicada call volume in each tree, with no relationship between trees because cicadas could not yet hear other cicadas in other trees.

For the third part of the simulation (5000 to 7500 seconds) we introduced interactions between locations. Trees were taken to be spaced in a 5-by-5 grid with one unit of spacing between nearest neighbors, so the maximum inter-tree distance was $\sqrt{32}$ units. In each tree the sound volume V_i was increased by a sum of terms $\sum_j u_{ij}$ proportional to the fraction of cicadas calling in every other tree v_j, subject to an inverse square law which produced a 32-fold reduction in sound between the closest and the most distant trees, and including a term to represent attenuation of sound by absorption within the forest, with a coefficient $k = 0.1$ (set to produce a further approximately 2 times reduction in sound between the most distant trees). Thus the addition to volume V_i at tree i due to cicadas in tree j was $u_{ij} = 0.04 v_j e^{-k d_{ij}} / d_{ij}^2$ where d_{ij} is the distance (in units as above) between trees i and j. The cycling in all the trees now synchronized, with the peaks and troughs occurring at the same time in all trees.

For the final part of the simulation (7500 to 10000 seconds), we slightly increased the mean cycling rate of all the cicadas to $c = 0.0125$, simulating their common response to an environmental change. This increase in rate produced an increase in mean volume (as less time was spent not calling), cycling amplitude and spatial synchrony between the trees.

To investigate the implications of a higher mean cycling rate for the amplitude and spatial synchrony of the cycles, we ran the simulation with a range of periods (t_{step}/c) for the 'not-calling' part of the cycle, from 3.5 to 5.5 seconds, with the result that faster calling produced systematically greater synchrony, as observed in our empirical data.

As described in methods, three alternate simulations were run in which the component of sound volume arising from other trees was adjusted at each tree to represent: 1. Extreme attenuation; the components of volume u_{ij} due to cicadas in trees more than 1 unit away were set to zero. 2. 'Realistic' signal delay; the components of volume due to cicadas in other trees were systematically delayed (by the use of past rather than current values), assuming trees were 75m apart and the speed of sound is 330m/s. 3. Extreme signal delay; the delay was taken to be 10 times the 'realistic' value.

DRAFT

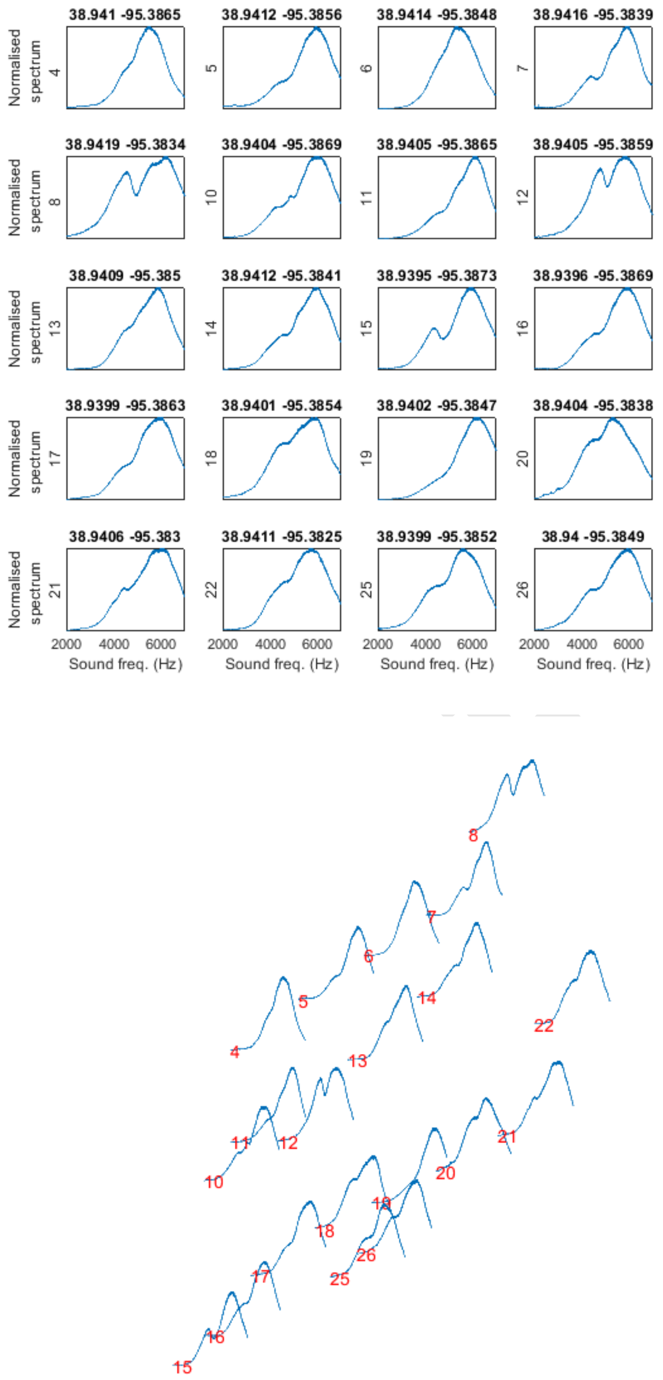

Fig. S1. Sound spectra associated with each point in space where recordings were made. The top 20 panels show the normalized spectra, their latitudes, longitudes and location numbers. The bottom sketch lays out the spectral curves according to their spatial positions. Wavelet transforms of the sound volume cycles are shown in Figs S2 to S21.

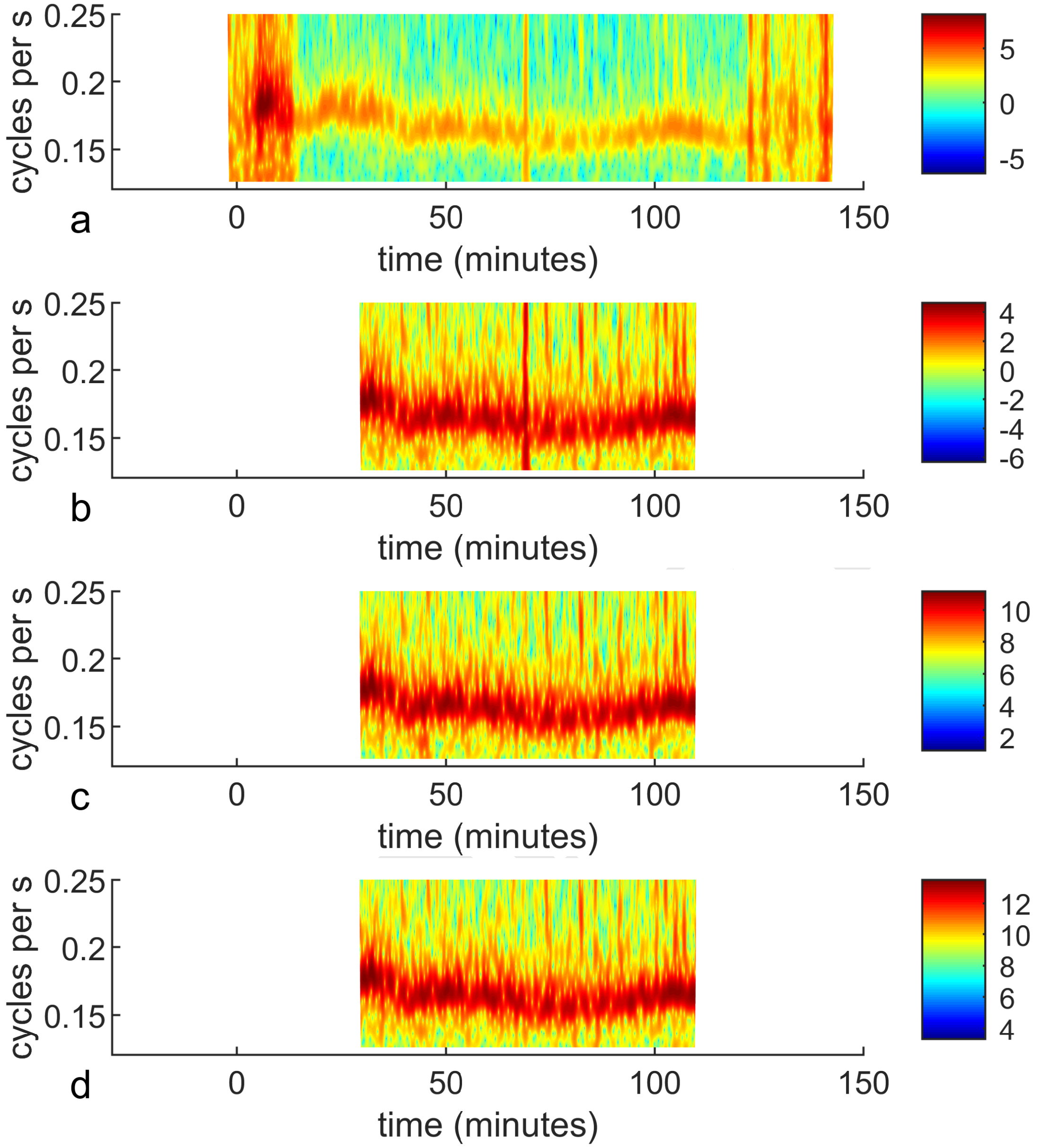

Fig. S2. Wavelet transform showing strong cycling of volume time series from the recording device at location 4 on Fig. S22, with period varying around approximately five seconds. Color indicates the log magnitude of fluctuations with a particular periodicity as this varies over time. a) Log magnitude of the transform of total sound volume in 0.1s windows through the whole signal, starting from the beginning of the recording. b) Log magnitude of the transform of total sound volume in 0.1s windows for useable data, i.e. during the period all devices were in place in the forest. c) Log magnitude of the transform of sound volume between 2000 and 7000 Hz (the frequencies of sound emitted by *M. cassini*) in 0.1s windows during the period all devices were in place in the forest. Volume between 2000 and 7000 Hz was obtained by band-pass filtering as described in SI Appendix, S2. d) Same as c, but volume between 2000 and 7000 Hz was obtained by matched filtering as described in SI Appendix, S2.

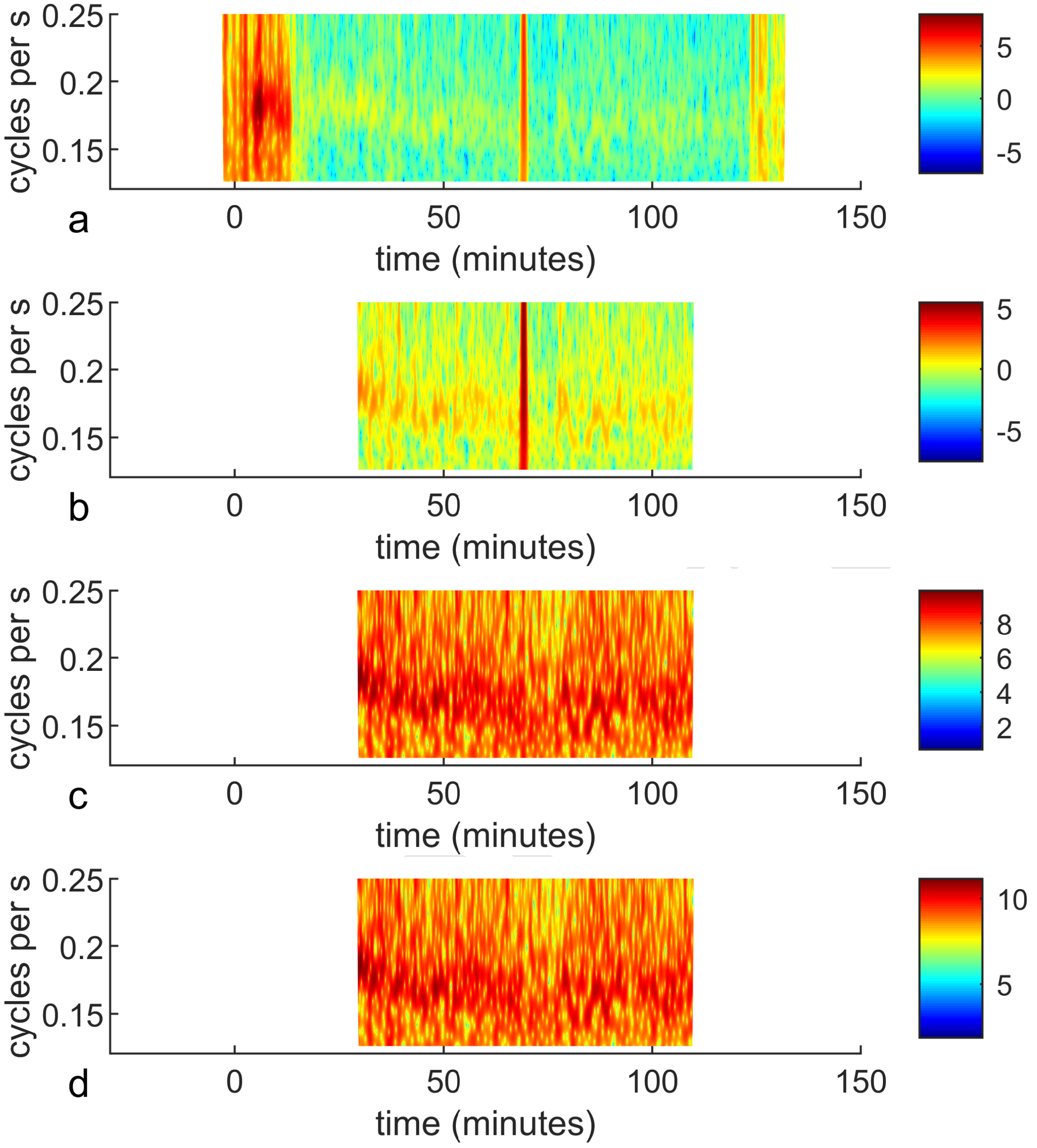

Fig. S3. Wavelet transform showing strong cycling of volume time series from the recording device at location 5 on Fig. S22, with period varying around approximately five seconds. This device also recorded video (brightness). Color indicates the log magnitude of fluctuations with a particular periodicity as this varies over time. a) Log magnitude of the transform of total sound volume in 0.1s windows through the whole signal, starting from the beginning of the recording. b) Log magnitude of the transform of total sound volume in 0.1s windows for useable data, i.e. during the period all devices were in place in the forest. c) Log magnitude of the transform of sound volume between 2000 and 7000 Hz (the frequencies of sound emitted by *M. cassini*) in 0.1s windows during the period all devices were in place in the forest. Volume between 2000 and 7000 Hz was obtained by band-pass filtering as described in SI Appendix, S2. d) Same as c, but volume between 2000 and 7000 Hz was obtained by matched filtering as described in SI Appendix, S2.

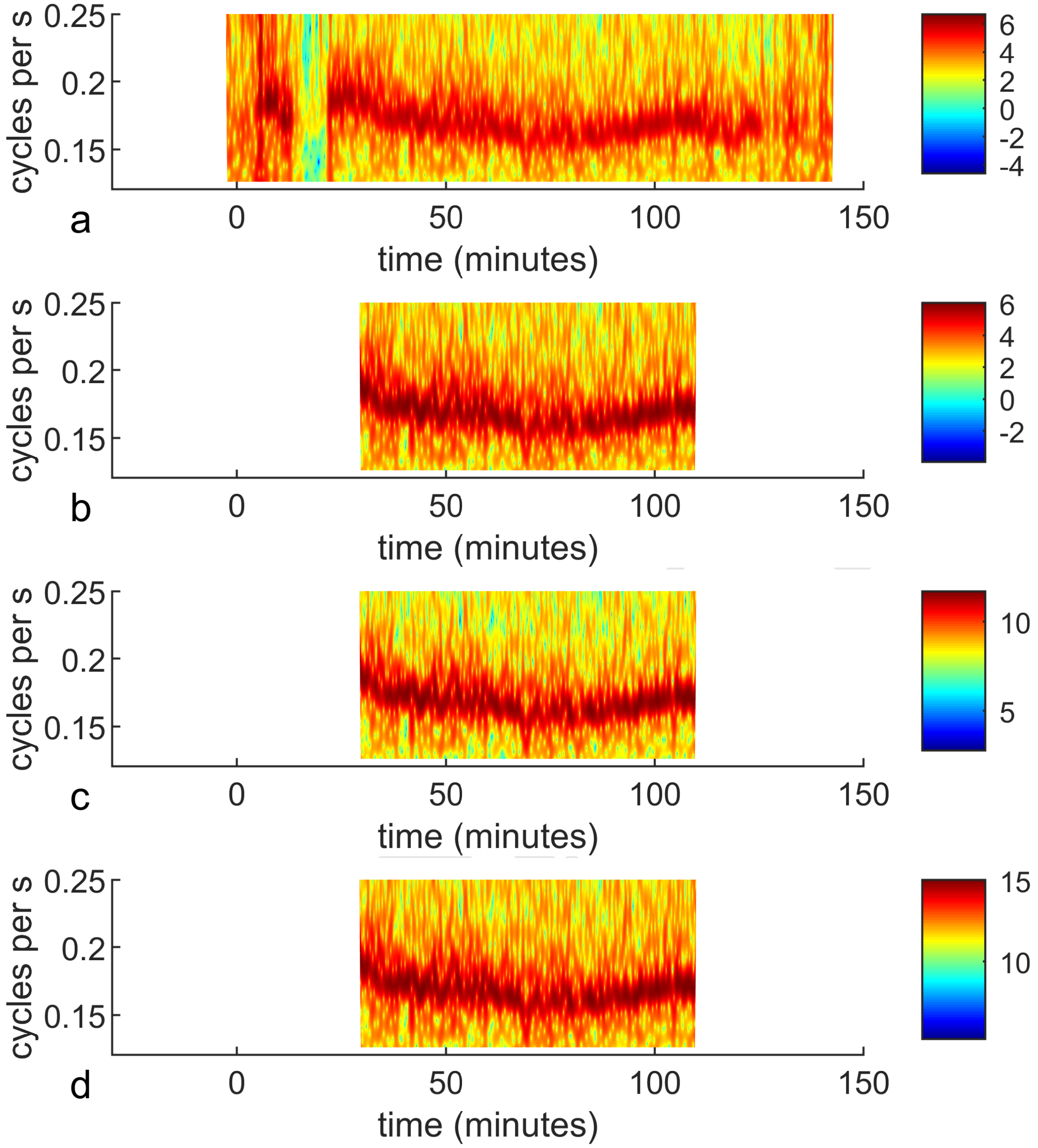

Fig. S4. Wavelet transform showing strong cycling of volume time series from the recording device at location 6 on Fig. S22, with period varying around approximately five seconds. Color indicates the log magnitude of fluctuations with a particular periodicity as this varies over time. a) Log magnitude of the transform of total sound volume in 0.1s windows through the whole signal, starting from the beginning of the recording. b) Log magnitude of the transform of total sound volume in 0.1s windows for useable data, i.e. during the period all devices were in place in the forest. c) Log magnitude of the transform of sound volume between 2000 and 7000 Hz (the frequencies of sound emitted by *M. cassini*) in 0.1s windows during the period all devices were in place in the forest. Volume between 2000 and 7000 Hz was obtained by band-pass filtering as described in SI Appendix, S2. d) Same as c, but volume between 2000 and 7000 Hz was obtained by matched filtering as described in SI Appendix, S2.

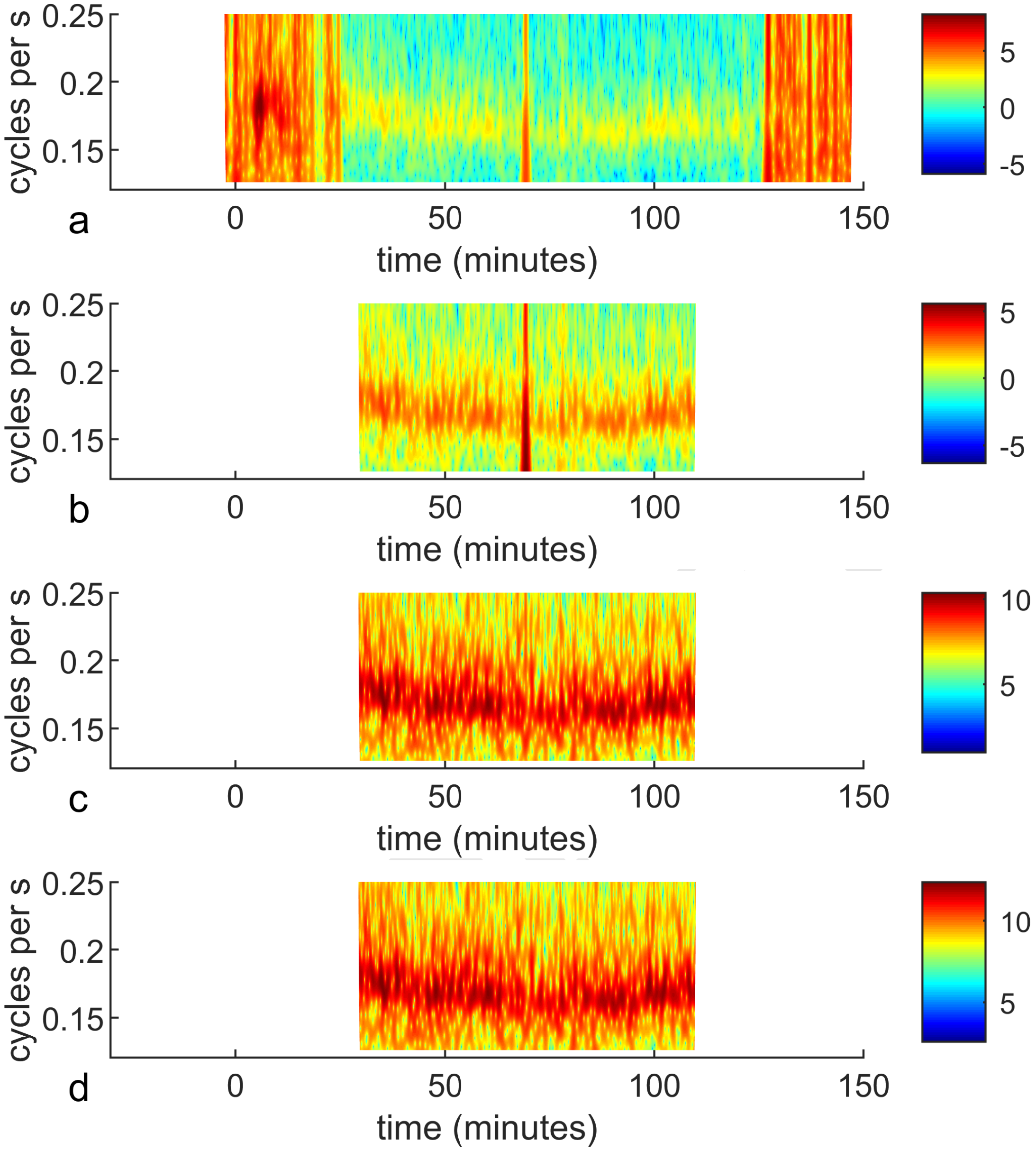

Fig. S5. Wavelet transform showing strong cycling of volume time series from the recording device at location 7 on Fig. S22, with period varying around approximately five seconds. Color indicates the log magnitude of fluctuations with a particular periodicity as this varies over time. a) Log magnitude of the transform of total sound volume in 0.1s windows through the whole signal, starting from the beginning of the recording. b) Log magnitude of the transform of total sound volume in 0.1s windows for useable data, i.e. during the period all devices were in place in the forest. c) Log magnitude of the transform of sound volume between 2000 and 7000 Hz (the frequencies of sound emitted by *M. cassini*) in 0.1s windows during the period all devices were in place in the forest. Volume between 2000 and 7000 Hz was obtained by band-pass filtering as described in SI Appendix, S2. d) Same as c, but volume between 2000 and 7000 Hz was obtained by matched filtering as described in SI Appendix, S2.

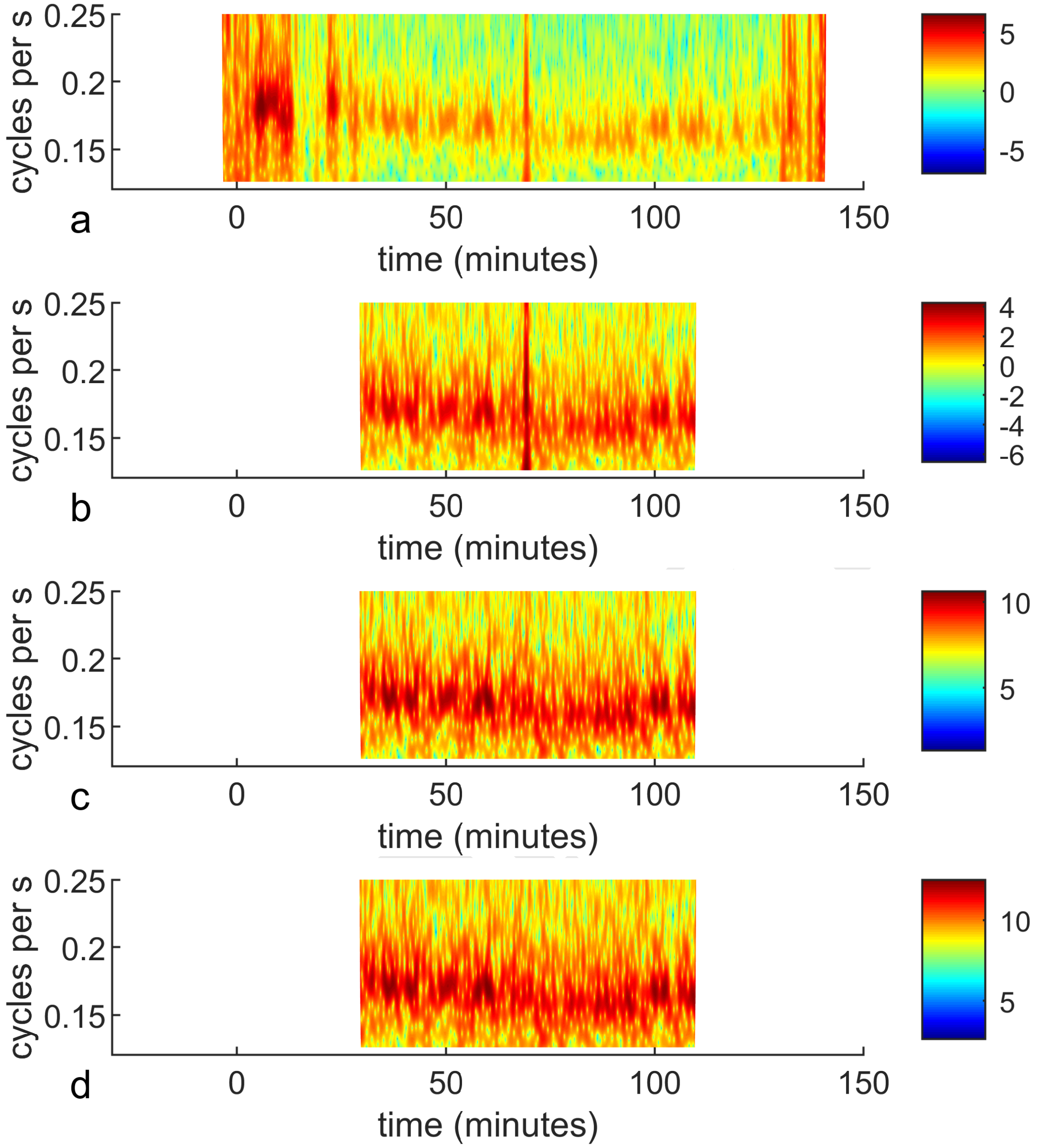

Fig. S6. Wavelet transform showing strong cycling of volume time series from the recording device at location 8 on Fig. S22, with period varying around approximately five seconds. Color indicates the log magnitude of fluctuations with a particular periodicity as this varies over time. a) Log magnitude of the transform of total sound volume in 0.1s windows through the whole signal, starting from the beginning of the recording. b) Log magnitude of the transform of total sound volume in 0.1s windows for useable data, i.e. during the period all devices were in place in the forest. c) Log magnitude of the transform of sound volume between 2000 and 7000 Hz (the frequencies of sound emitted by *M. cassini*) in 0.1s windows during the period all devices were in place in the forest. Volume between 2000 and 7000 Hz was obtained by band-pass filtering as described in SI Appendix, S2. d) Same as c, but volume between 2000 and 7000 Hz was obtained by matched filtering as described in SI Appendix, S2.

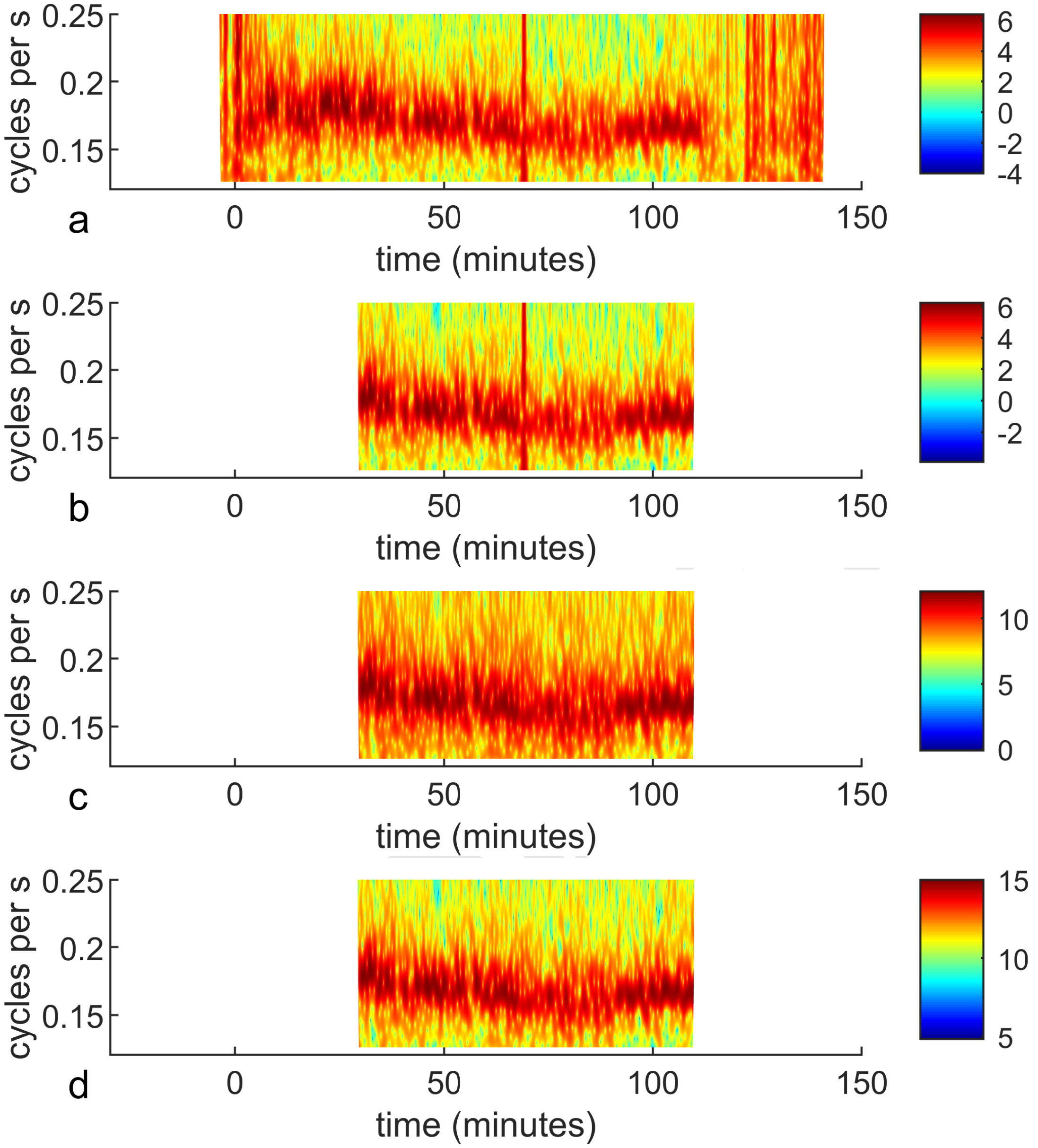

Fig. S7. Wavelet transform showing strong cycling of volume time series from the recording device at location 10 on Fig. S22, with period varying around approximately five seconds. Color indicates the log magnitude of fluctuations with a particular periodicity as this varies over time. a) Log magnitude of the transform of total sound volume in 0.1s windows through the whole signal, starting from the beginning of the recording. b) Log magnitude of the transform of total sound volume in 0.1s windows for useable data, i.e. during the period all devices were in place in the forest. c) Log magnitude of the transform of sound volume between 2000 and 7000 Hz (the frequencies of sound emitted by *M. cassini*) in 0.1s windows during the period all devices were in place in the forest. Volume between 2000 and 7000 Hz was obtained by band-pass filtering as described in SI Appendix, S2. d) Same as c, but volume between 2000 and 7000 Hz was obtained by matched filtering as described in SI Appendix, S2.

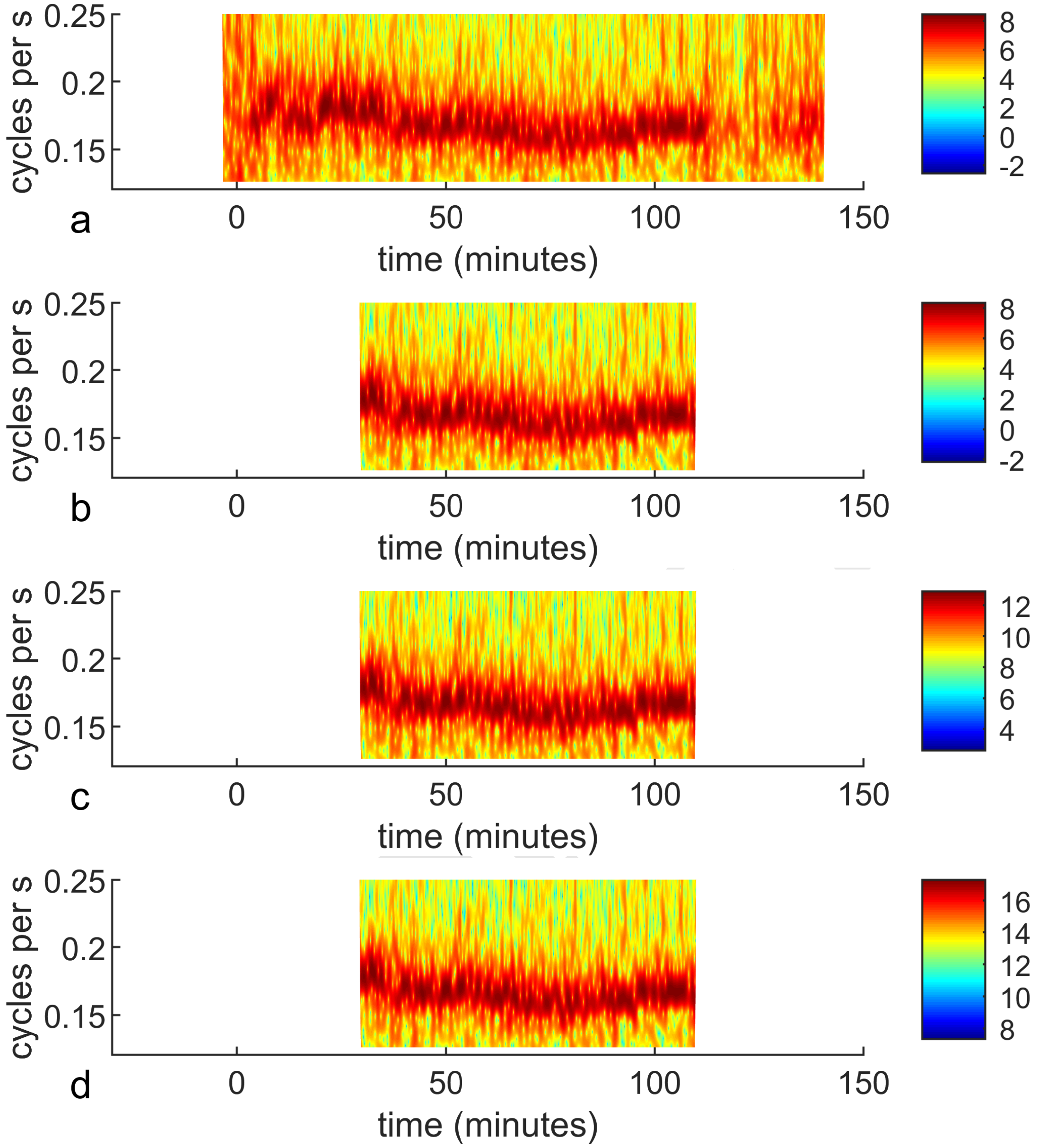

Fig. S8. Wavelet transform showing strong cycling of volume time series from the recording device at location 11 on Fig. S22, with period varying around approximately five seconds. Color indicates the log magnitude of fluctuations with a particular periodicity as this varies over time. a) Log magnitude of the transform of total sound volume in 0.1s windows through the whole signal, starting from the beginning of the recording. b) Log magnitude of the transform of total sound volume in 0.1s windows for useable data, i.e. during the period all devices were in place in the forest. c) Log magnitude of the transform of sound volume between 2000 and 7000 Hz (the frequencies of sound emitted by *M. cassini*) in 0.1s windows during the period all devices were in place in the forest. Volume between 2000 and 7000 Hz was obtained by band-pass filtering as described in SI Appendix, S2. d) Same as c, but volume between 2000 and 7000 Hz was obtained by matched filtering as described in SI Appendix, S2.

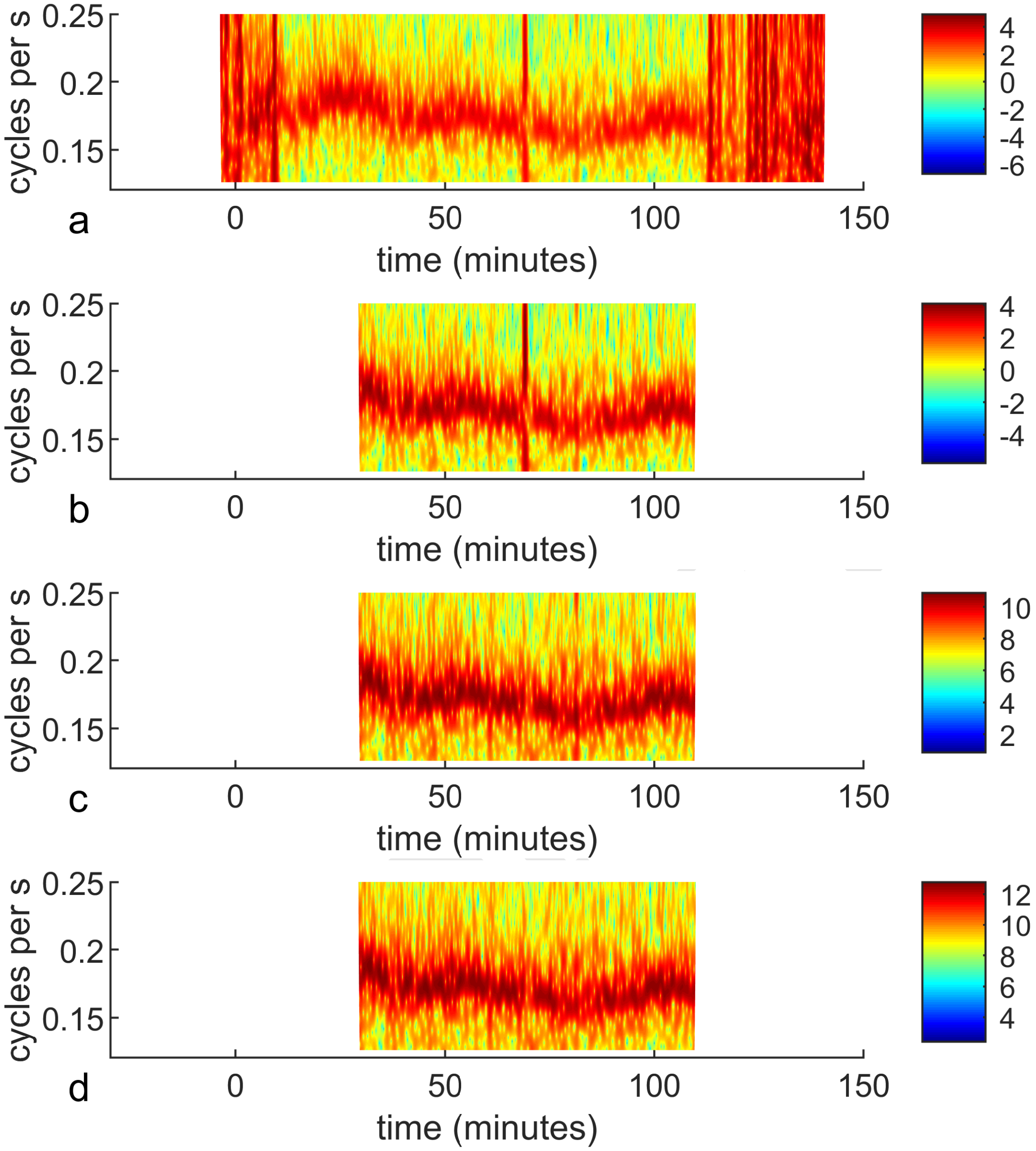

Fig. S9. Wavelet transform showing strong cycling of volume time series from the recording device at location 12 on Fig. S22, with period varying around approximately five seconds. Color indicates the log magnitude of fluctuations with a particular periodicity as this varies over time. a) Log magnitude of the transform of total sound volume in 0.1s windows through the whole signal, starting from the beginning of the recording. b) Log magnitude of the transform of total sound volume in 0.1s windows for useable data, i.e. during the period all devices were in place in the forest. c) Log magnitude of the transform of sound volume between 2000 and 7000 Hz (the frequencies of sound emitted by *M. cassini*) in 0.1s windows during the period all devices were in place in the forest. Volume between 2000 and 7000 Hz was obtained by band-pass filtering as described in SI Appendix, S2. d) Same as c, but volume between 2000 and 7000 Hz was obtained by matched filtering as described in SI Appendix, S2.

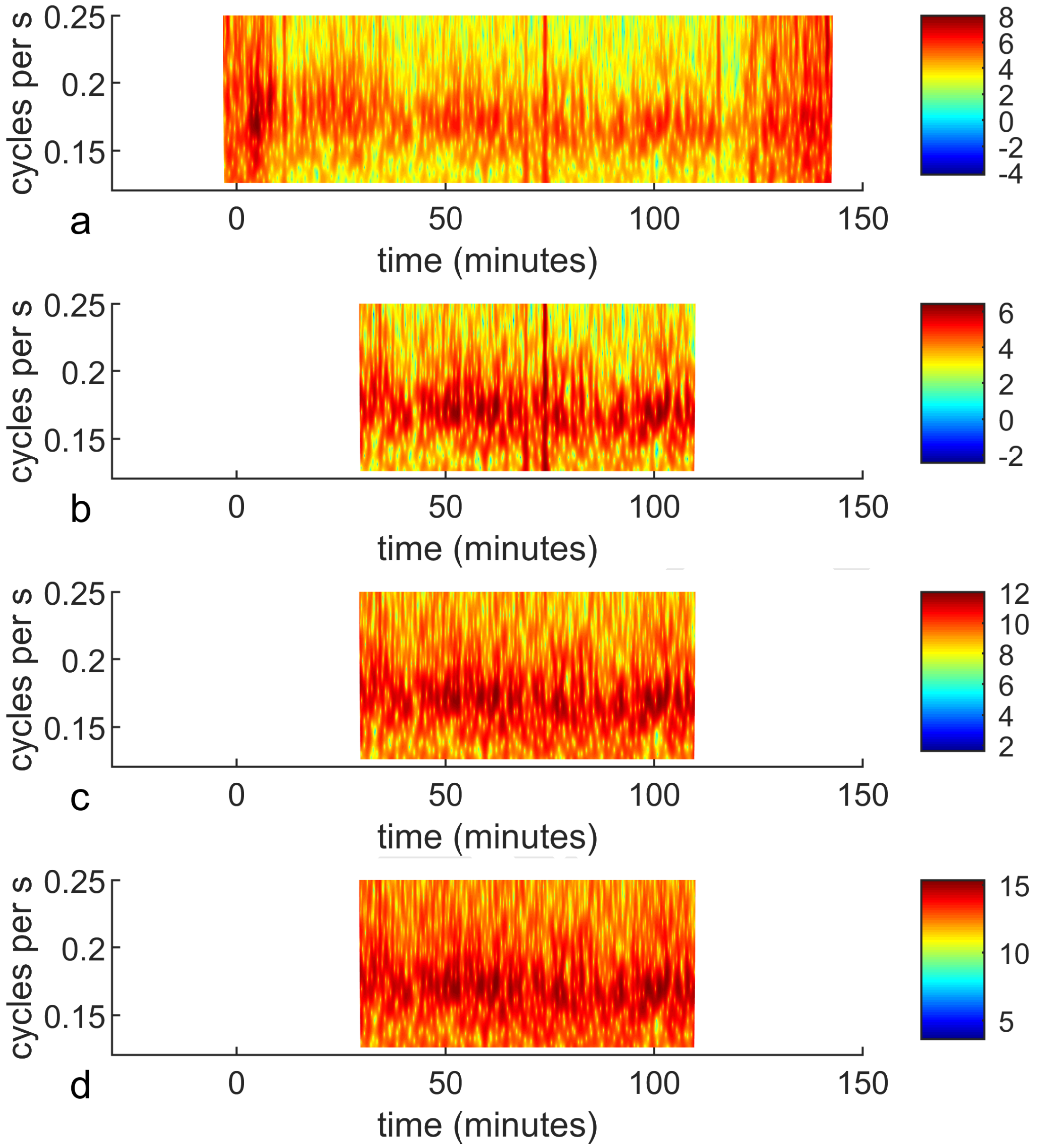

Fig. S10. Wavelet transform showing strong cycling of volume time series from the recording device at location 13 on Fig. S22, with period varying around approximately five seconds. Color indicates the log magnitude of fluctuations with a particular periodicity as this varies over time. a) Log magnitude of the transform of total sound volume in 0.1s windows through the whole signal, starting from the beginning of the recording. b) Log magnitude of the transform of total sound volume in 0.1s windows for useable data, i.e. during the period all devices were in place in the forest. c) Log magnitude of the transform of sound volume between 2000 and 7000 Hz (the frequencies of sound emitted by *M. cassini*) in 0.1s windows during the period all devices were in place in the forest. Volume between 2000 and 7000 Hz was obtained by band-pass filtering as described in SI Appendix, S2. d) Same as c, but volume between 2000 and 7000 Hz was obtained by matched filtering as described in SI Appendix, S2.

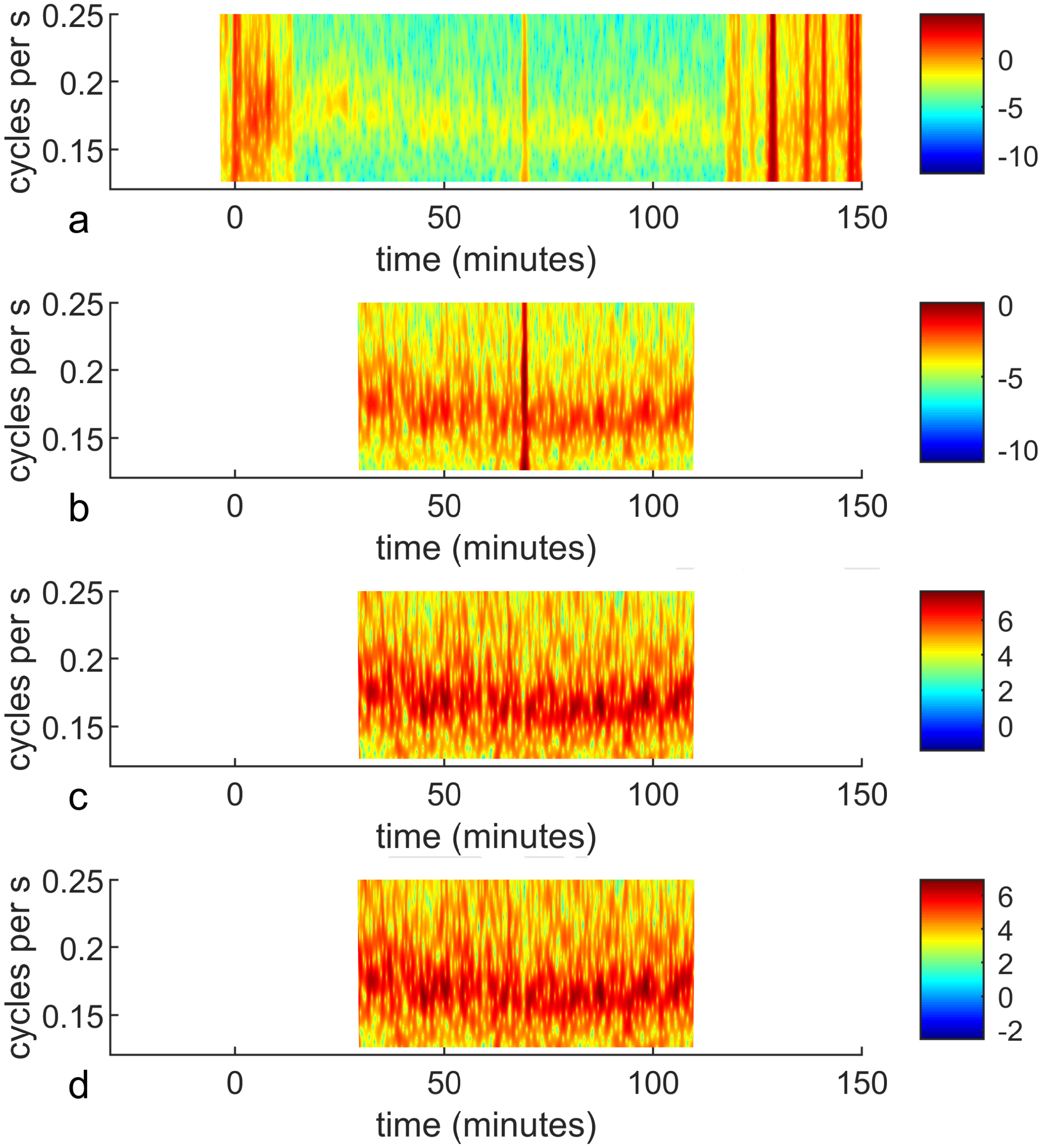

Fig. S11. Wavelet transform showing strong cycling of volume time series from the recording device at location 14 on Fig. S22, with period varying around approximately five seconds. Color indicates the log magnitude of fluctuations with a particular periodicity as this varies over time. a) Log magnitude of the transform of total sound volume in 0.1s windows through the whole signal, starting from the beginning of the recording. b) Log magnitude of the transform of total sound volume in 0.1s windows for useable data, i.e. during the period all devices were in place in the forest. c) Log magnitude of the transform of sound volume between 2000 and 7000 Hz (the frequencies of sound emitted by *M. cassini*) in 0.1s windows during the period all devices were in place in the forest. Volume between 2000 and 7000 Hz was obtained by band-pass filtering as described in SI Appendix, S2. d) Same as c, but volume between 2000 and 7000 Hz was obtained by matched filtering as described in SI Appendix, S2.

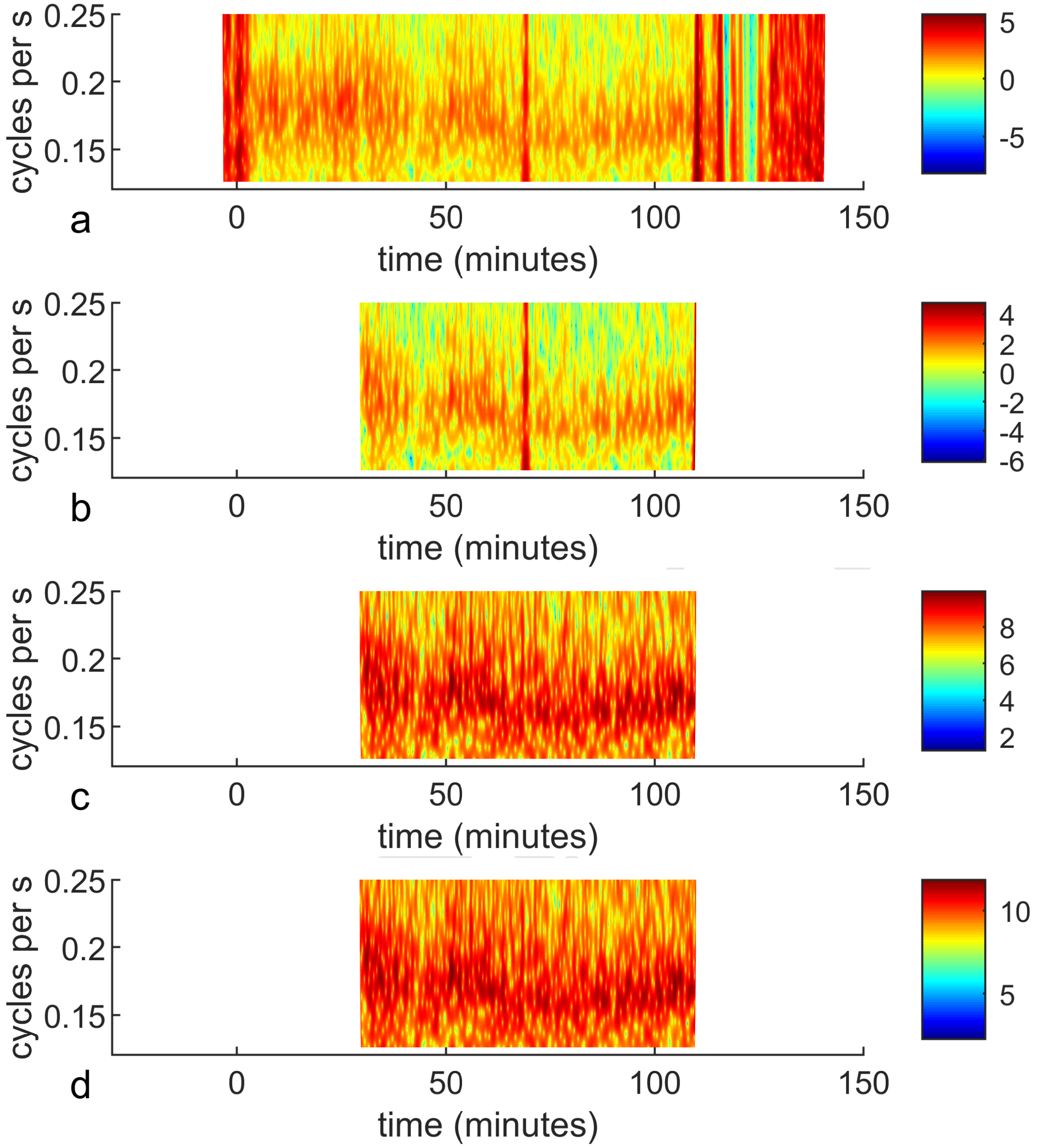

Fig. S12. Wavelet transform showing strong cycling of volume time series from the recording device at location 15 on Fig. S22, with period varying around approximately five seconds. Color indicates the log magnitude of fluctuations with a particular periodicity as this varies over time. a) Log magnitude of the transform of total sound volume in 0.1s windows through the whole signal, starting from the beginning of the recording. b) Log magnitude of the transform of total sound volume in 0.1s windows for useable data, i.e. during the period all devices were in place in the forest. c) Log magnitude of the transform of sound volume between 2000 and 7000 Hz (the frequencies of sound emitted by *M. cassini*) in 0.1s windows during the period all devices were in place in the forest. Volume between 2000 and 7000 Hz was obtained by band-pass filtering as described in SI Appendix, S2. d) Same as c, but volume between 2000 and 7000 Hz was obtained by matched filtering as described in SI Appendix, S2.

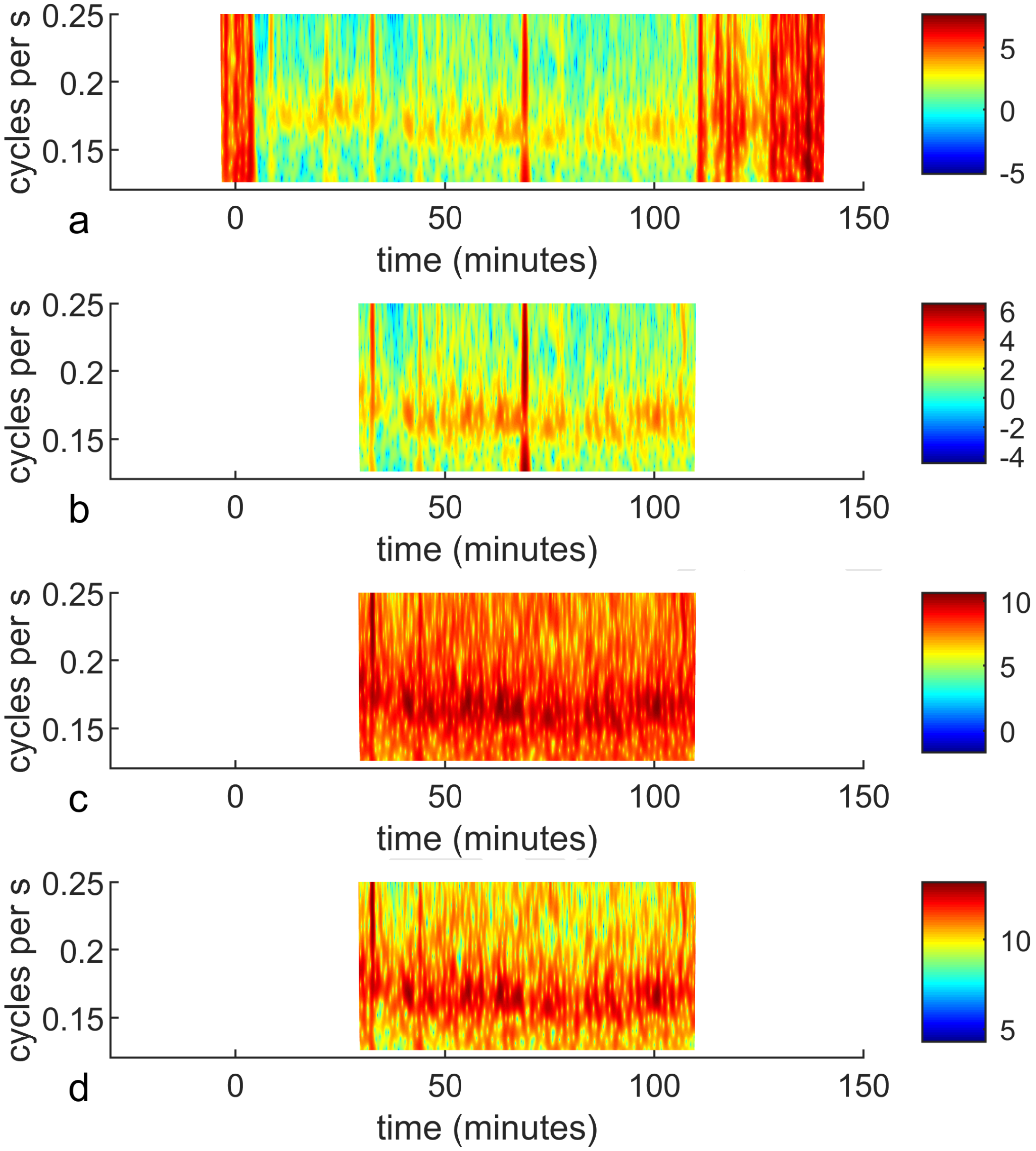

Fig. S13. Wavelet transform showing strong cycling of volume time series from the recording device at location 16 on Fig. S22, with period varying around approximately five seconds. Color indicates the log magnitude of fluctuations with a particular periodicity as this varies over time. a) Log magnitude of the transform of total sound volume in 0.1s windows through the whole signal, starting from the beginning of the recording. b) Log magnitude of the transform of total sound volume in 0.1s windows for useable data, i.e. during the period all devices were in place in the forest. c) Log magnitude of the transform of sound volume between 2000 and 7000 Hz (the frequencies of sound emitted by *M. cassini*) in 0.1s windows during the period all devices were in place in the forest. Volume between 2000 and 7000 Hz was obtained by band-pass filtering as described in SI Appendix, S2. d) Same as c, but volume between 2000 and 7000 Hz was obtained by matched filtering as described in SI Appendix, S2.

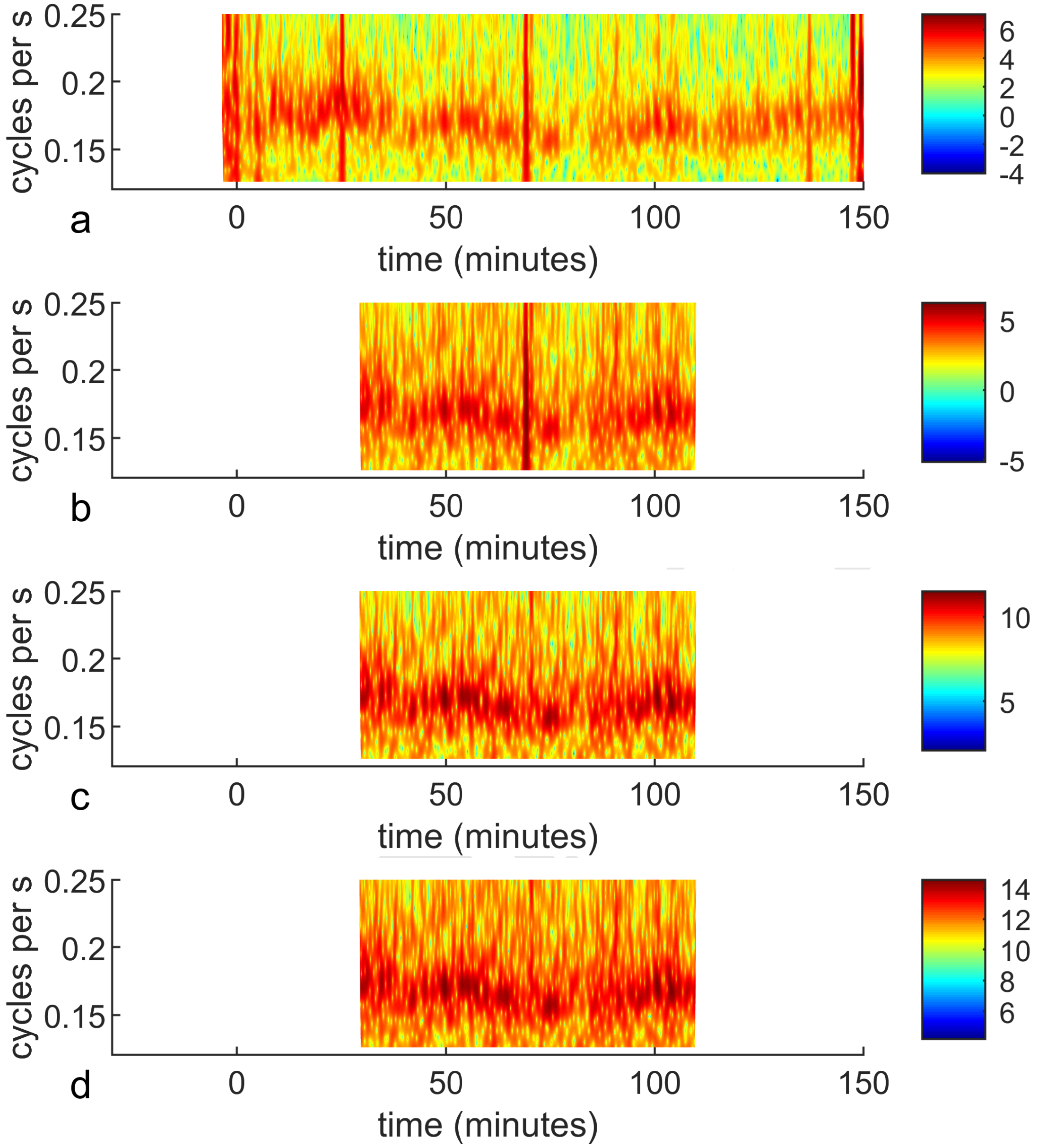

Fig. S14. Wavelet transform showing strong cycling of volume time series from the recording device at location 17 on Fig. S22, with period varying around approximately five seconds. Color indicates the log magnitude of fluctuations with a particular periodicity as this varies over time. a) Log magnitude of the transform of total sound volume in 0.1s windows through the whole signal, starting from the beginning of the recording. b) Log magnitude of the transform of total sound volume in 0.1s windows for useable data, i.e. during the period all devices were in place in the forest. c) Log magnitude of the transform of sound volume between 2000 and 7000 Hz (the frequencies of sound emitted by *M. cassini*) in 0.1s windows during the period all devices were in place in the forest. Volume between 2000 and 7000 Hz was obtained by band-pass filtering as described in SI Appendix, S2. d) Same as c, but volume between 2000 and 7000 Hz was obtained by matched filtering as described in SI Appendix, S2.

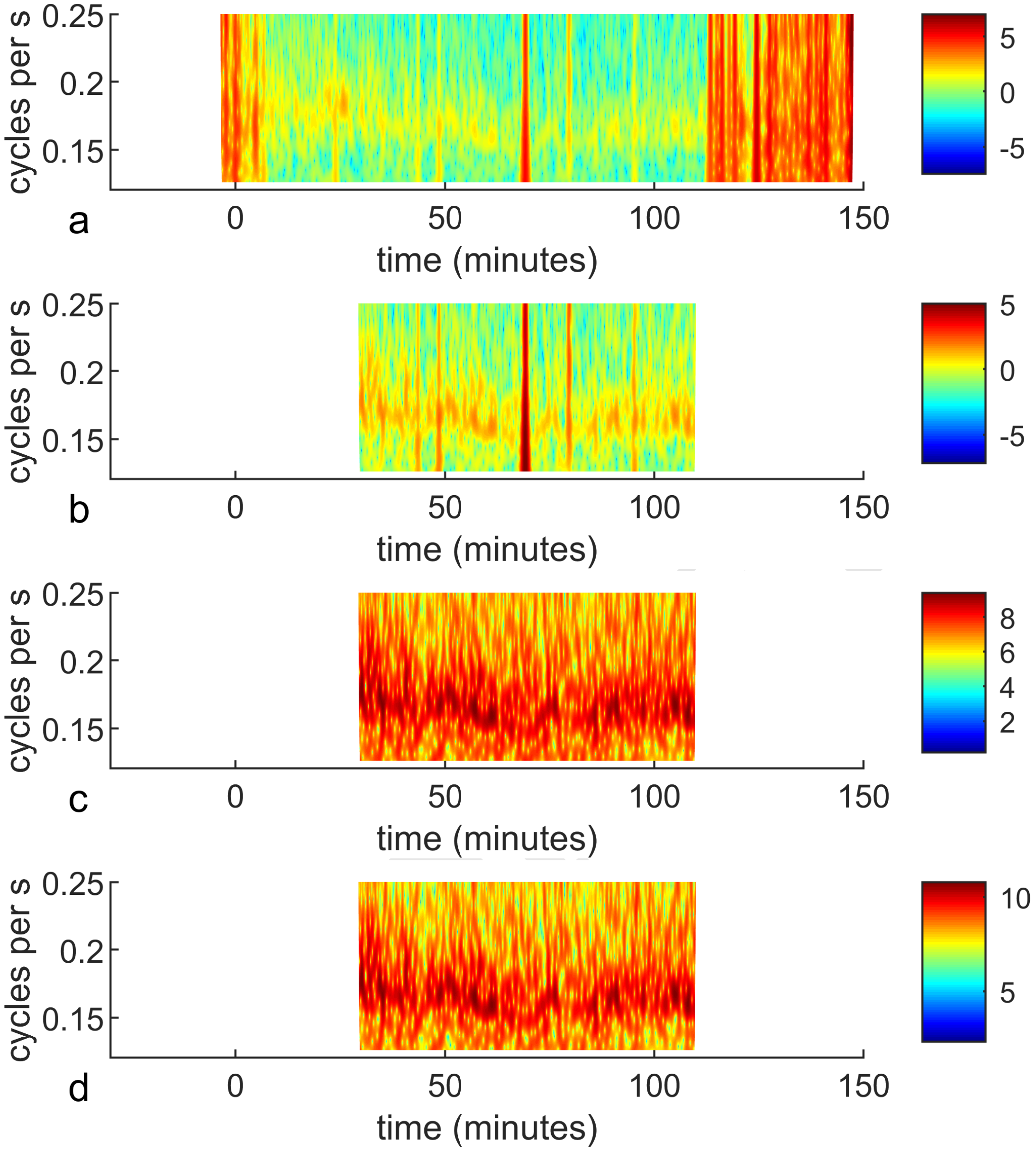

Fig. S15. Wavelet transform showing strong cycling of volume time series from the recording device at location 18 on Fig. S22, with period varying around approximately five seconds. Color indicates the log magnitude of fluctuations with a particular periodicity as this varies over time. a) Log magnitude of the transform of total sound volume in 0.1s windows through the whole signal, starting from the beginning of the recording. b) Log magnitude of the transform of total sound volume in 0.1s windows for useable data, i.e. during the period all devices were in place in the forest. c) Log magnitude of the transform of sound volume between 2000 and 7000 Hz (the frequencies of sound emitted by *M. cassini*) in 0.1s windows during the period all devices were in place in the forest. Volume between 2000 and 7000 Hz was obtained by band-pass filtering as described in SI Appendix, S2. d) Same as c, but volume between 2000 and 7000 Hz was obtained by matched filtering as described in SI Appendix, S2.

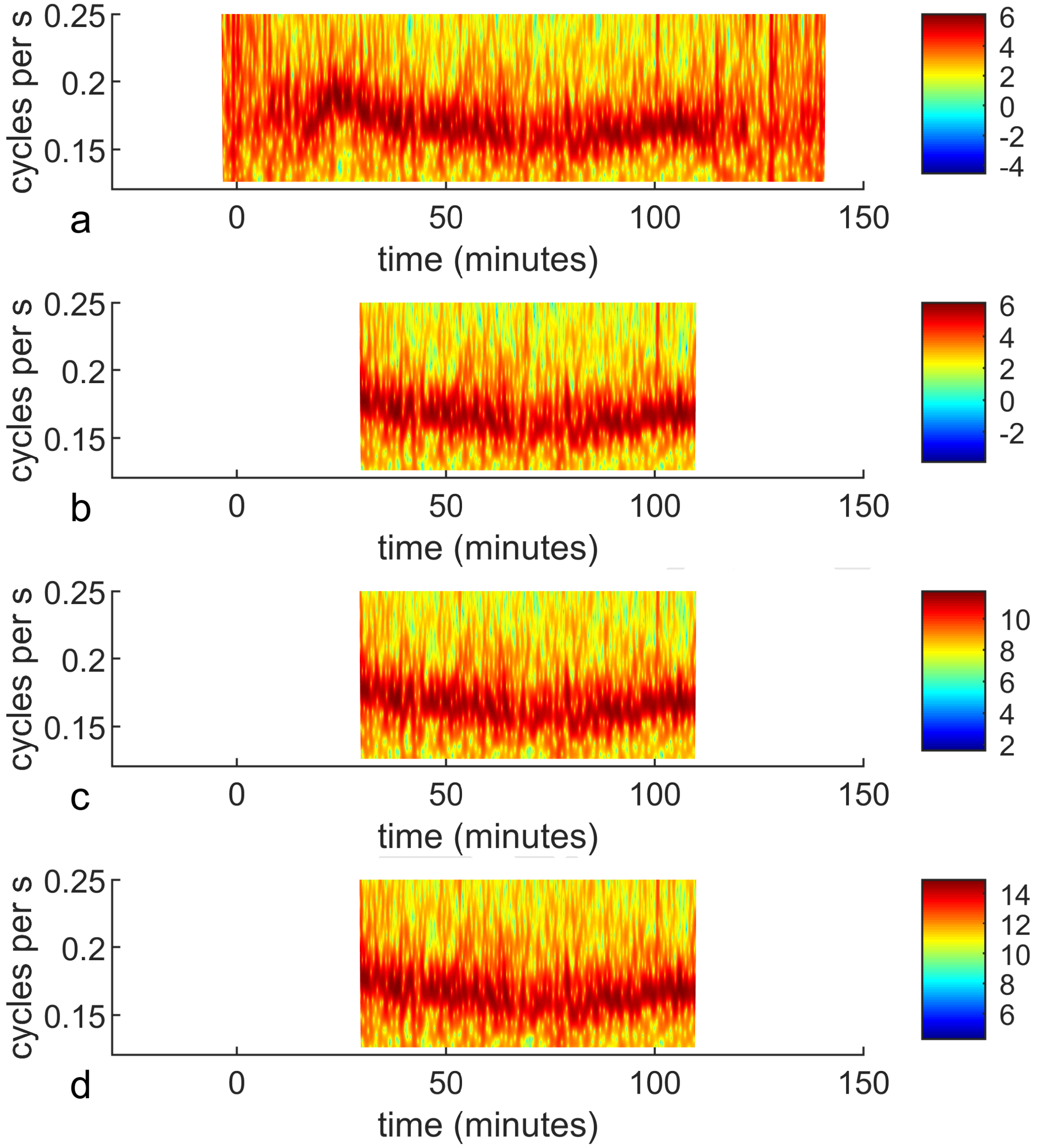

Fig. S16. Wavelet transform showing strong cycling of volume time series from the recording device at location 19 on Fig. S22, with period varying around approximately five seconds. Color indicates the log magnitude of fluctuations with a particular periodicity as this varies over time. a) Log magnitude of the transform of total sound volume in 0.1s windows through the whole signal, starting from the beginning of the recording. b) Log magnitude of the transform of total sound volume in 0.1s windows for useable data, i.e. during the period all devices were in place in the forest. c) Log magnitude of the transform of sound volume between 2000 and 7000 Hz (the frequencies of sound emitted by *M. cassini*) in 0.1s windows during the period all devices were in place in the forest. Volume between 2000 and 7000 Hz was obtained by band-pass filtering as described in SI Appendix, S2. d) Same as c, but volume between 2000 and 7000 Hz was obtained by matched filtering as described in SI Appendix, S2.

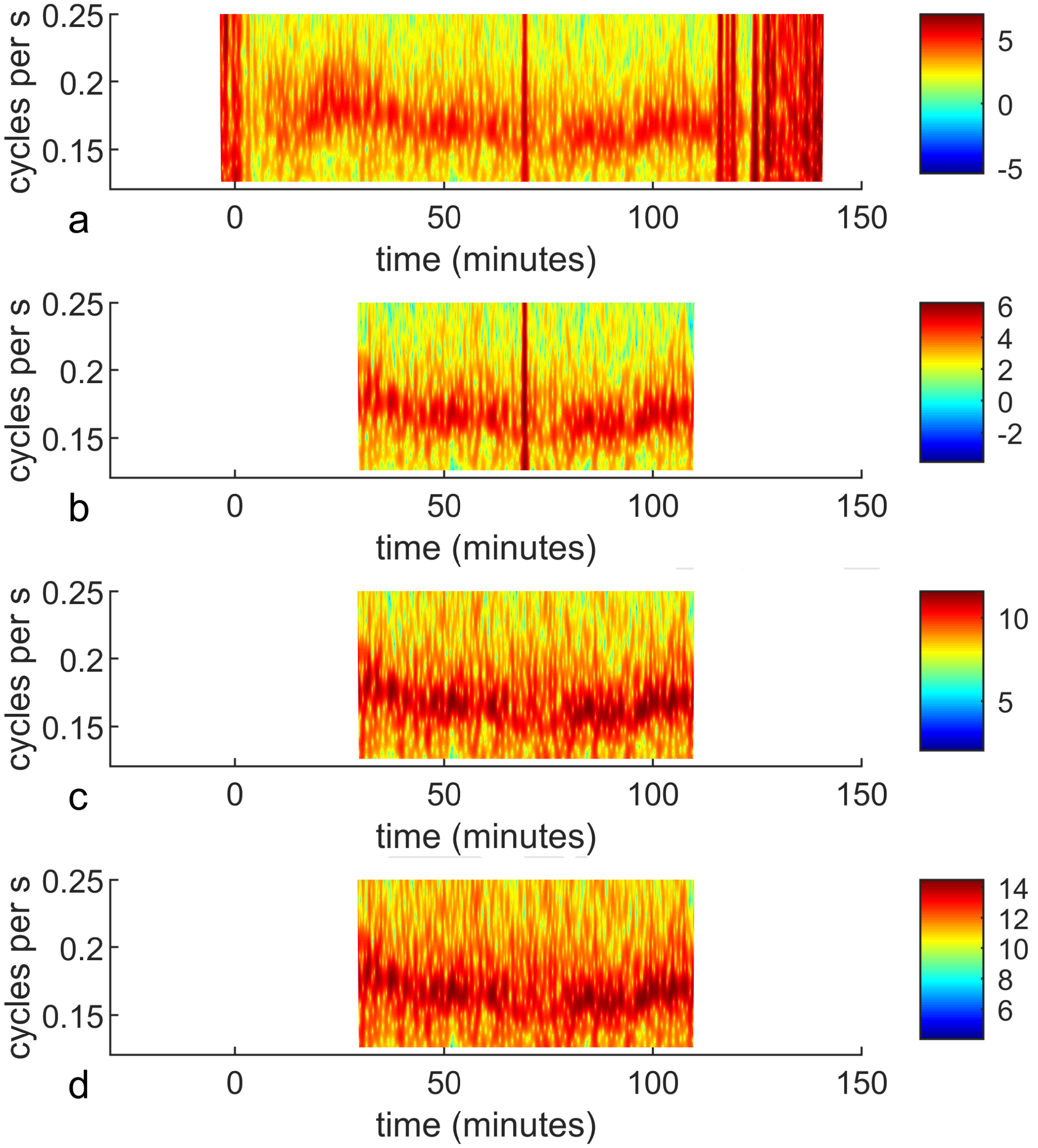

Fig. S17. Wavelet transform showing strong cycling of volume time series from the recording device at location 20 on Fig. S22, with period varying around approximately five seconds. Color indicates the log magnitude of fluctuations with a particular periodicity as this varies over time. a) Log magnitude of the transform of total sound volume in 0.1s windows through the whole signal, starting from the beginning of the recording. b) Log magnitude of the transform of total sound volume in 0.1s windows for useable data, i.e. during the period all devices were in place in the forest. c) Log magnitude of the transform of sound volume between 2000 and 7000 Hz (the frequencies of sound emitted by *M. cassini*) in 0.1s windows during the period all devices were in place in the forest. Volume between 2000 and 7000 Hz was obtained by band-pass filtering as described in SI Appendix, S2. d) Same as c, but volume between 2000 and 7000 Hz was obtained by matched filtering as described in SI Appendix, S2.

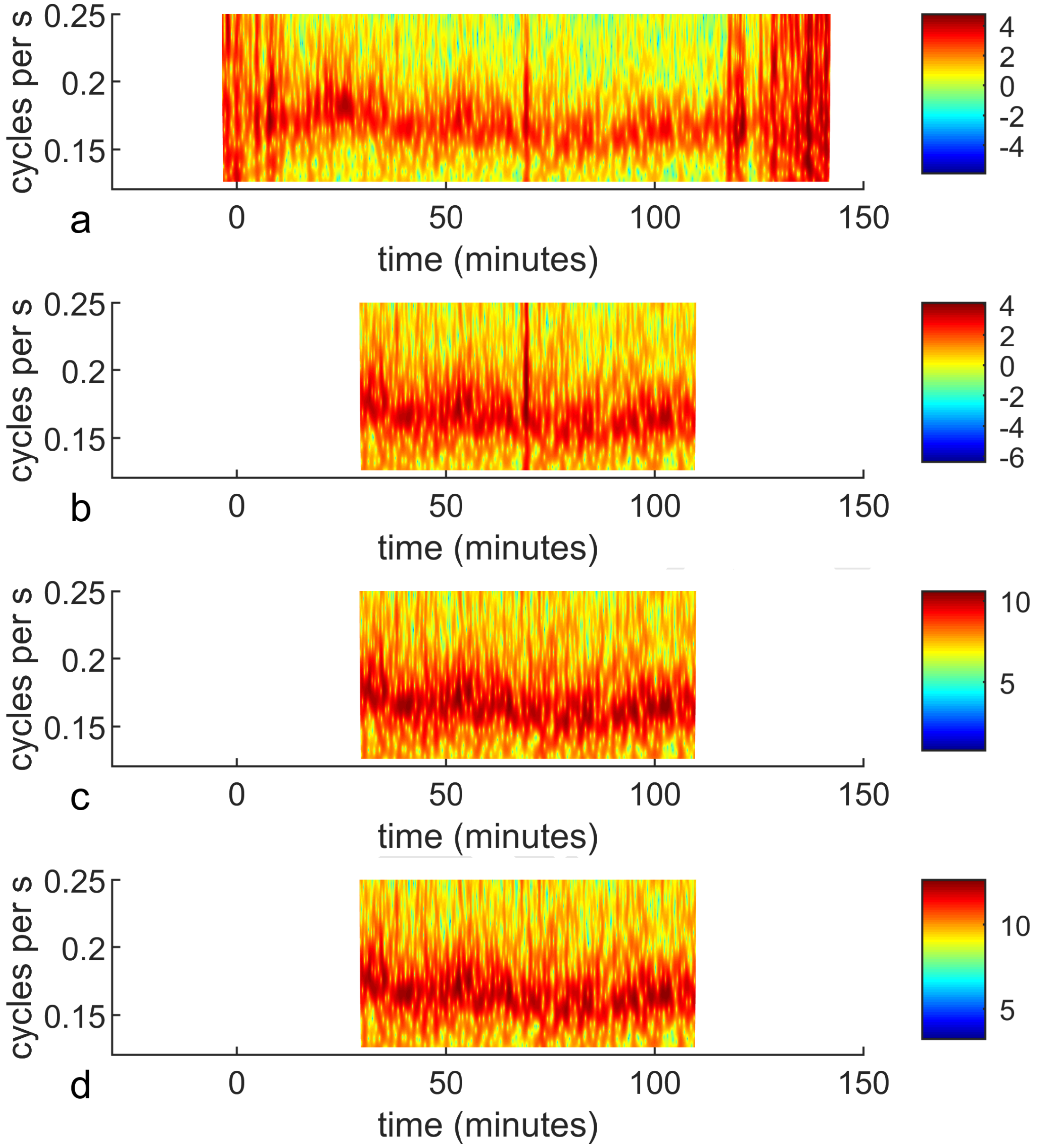

Fig. S18. Wavelet transform showing strong cycling of volume time series from the recording device at location 21 on Fig. S22, with period varying around approximately five seconds. Color indicates the log magnitude of fluctuations with a particular periodicity as this varies over time. a) Log magnitude of the transform of total sound volume in 0.1s windows through the whole signal, starting from the beginning of the recording. b) Log magnitude of the transform of total sound volume in 0.1s windows for useable data, i.e. during the period all devices were in place in the forest. c) Log magnitude of the transform of sound volume between 2000 and 7000 Hz (the frequencies of sound emitted by *M. cassini*) in 0.1s windows during the period all devices were in place in the forest. Volume between 2000 and 7000 Hz was obtained by band-pass filtering as described in SI Appendix, S2. d) Same as c, but volume between 2000 and 7000 Hz was obtained by matched filtering as described in SI Appendix, S2.

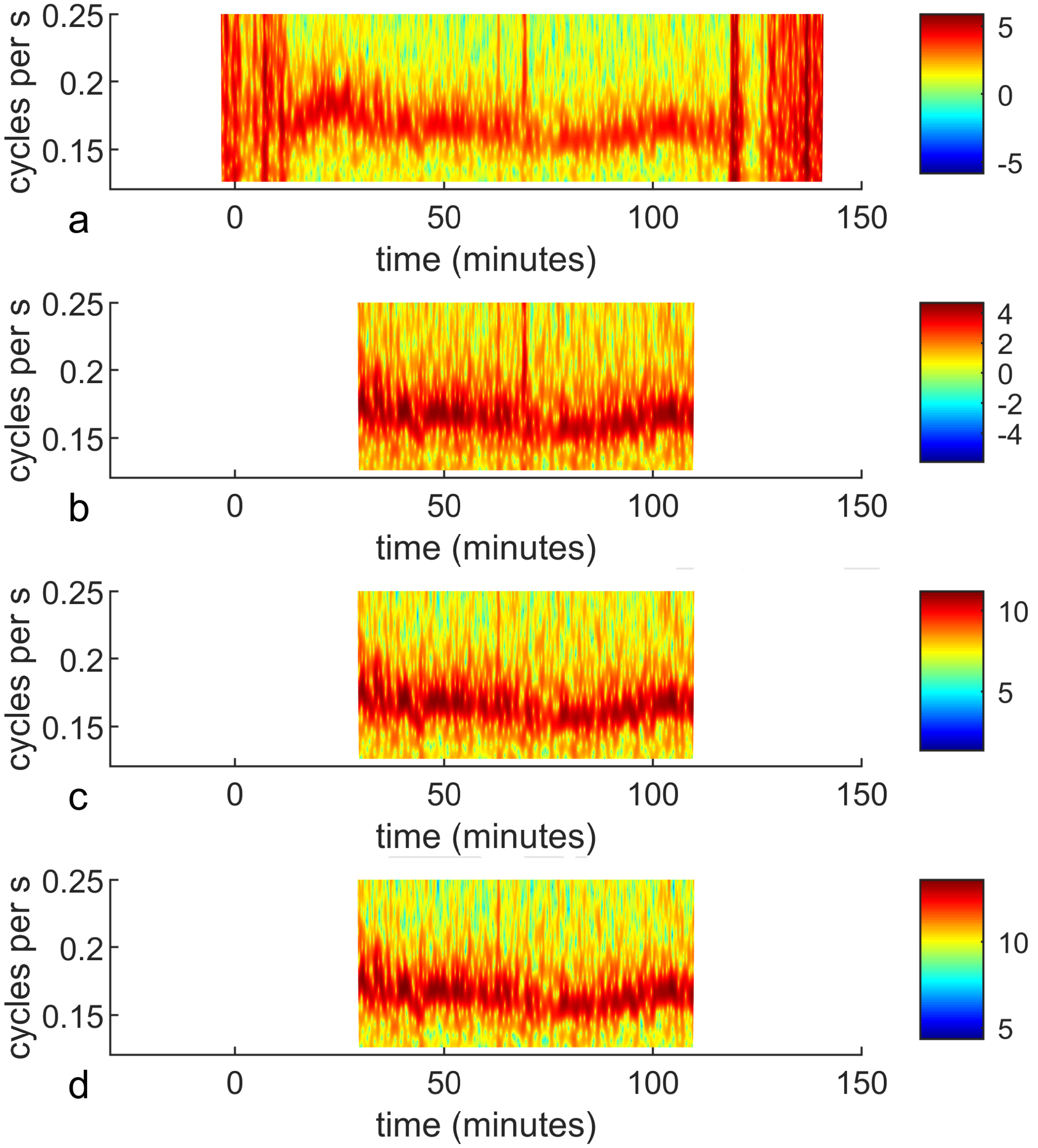

Fig. S19. Wavelet transform showing strong cycling of volume time series from the recording device at location 22 on Fig. S22, with period varying around approximately five seconds. Color indicates the log magnitude of fluctuations with a particular periodicity as this varies over time. a) Log magnitude of the transform of total sound volume in 0.1s windows through the whole signal, starting from the beginning of the recording. b) Log magnitude of the transform of total sound volume in 0.1s windows for useable data, i.e. during the period all devices were in place in the forest. c) Log magnitude of the transform of sound volume between 2000 and 7000 Hz (the frequencies of sound emitted by *M. cassini*) in 0.1s windows during the period all devices were in place in the forest. Volume between 2000 and 7000 Hz was obtained by band-pass filtering as described in SI Appendix, S2. d) Same as c, but volume between 2000 and 7000 Hz was obtained by matched filtering as described in SI Appendix, S2.

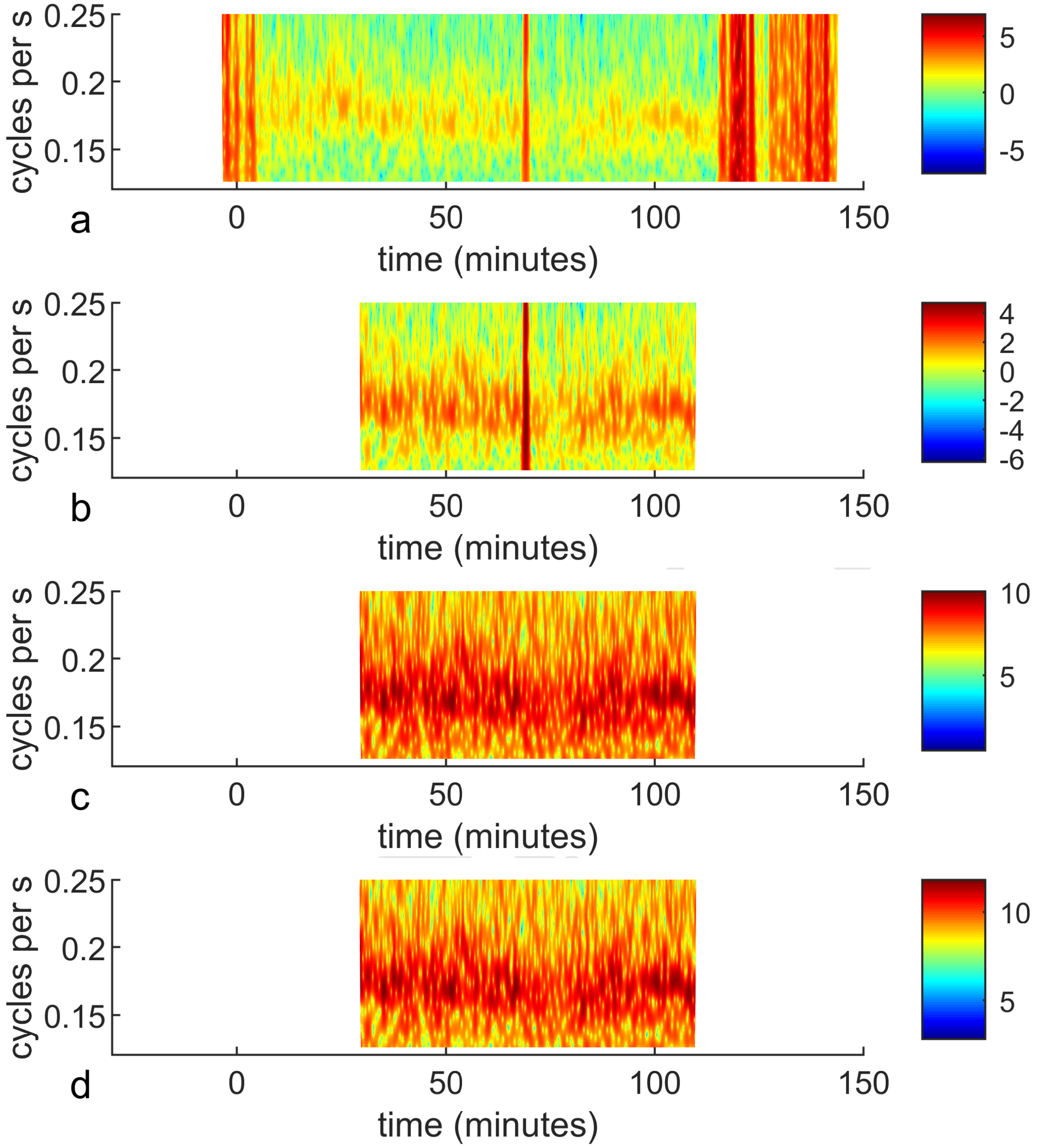

Fig. S20. Wavelet transform showing strong cycling of volume time series from the recording device at location 25 on Fig. S22, with period varying around approximately five seconds. Color indicates the log magnitude of fluctuations with a particular periodicity as this varies over time. a) Log magnitude of the transform of total sound volume in 0.1s windows through the whole signal, starting from the beginning of the recording. b) Log magnitude of the transform of total sound volume in 0.1s windows for useable data, i.e. during the period all devices were in place in the forest. c) Log magnitude of the transform of sound volume between 2000 and 7000 Hz (the frequencies of sound emitted by *M. cassini*) in 0.1s windows during the period all devices were in place in the forest. Volume between 2000 and 7000 Hz was obtained by band-pass filtering as described in SI Appendix, S2. d) Same as c, but volume between 2000 and 7000 Hz was obtained by matched filtering as described in SI Appendix, S2.

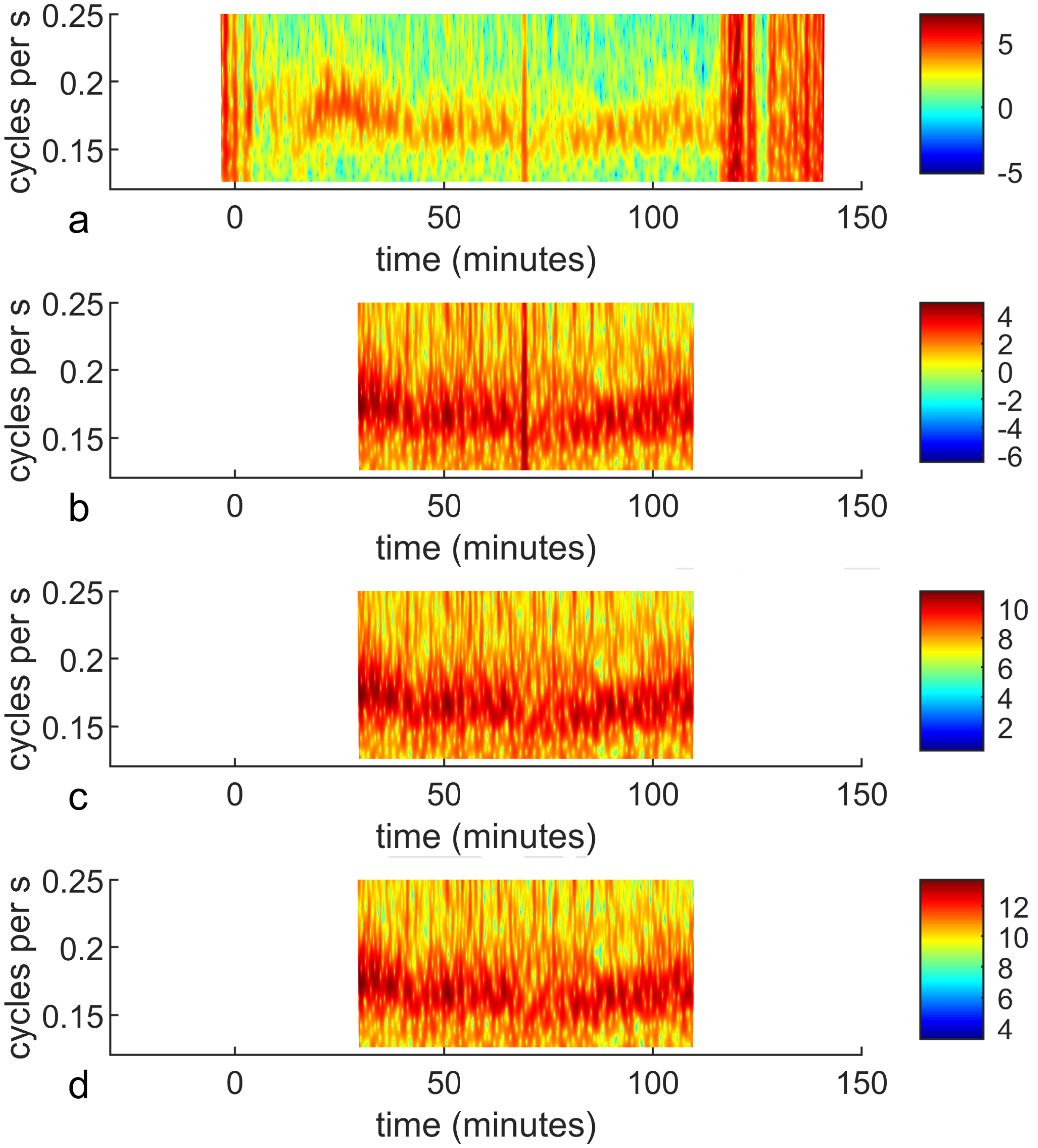

Fig. S21. Wavelet transform showing strong cycling of volume time series from the recording device at location 26 on Fig. S22, with period varying around approximately five seconds. Color indicates the log magnitude of fluctuations with a particular periodicity as this varies over time. a) Log magnitude of the transform of total sound volume in 0.1s windows through the whole signal, starting from the beginning of the recording. b) Log magnitude of the transform of total sound volume in 0.1s windows for useable data, i.e. during the period all devices were in place in the forest. c) Log magnitude of the transform of sound volume between 2000 and 7000 Hz (the frequencies of sound emitted by *M. cassini*) in 0.1s windows during the period all devices were in place in the forest. Volume between 2000 and 7000 Hz was obtained by band-pass filtering as described in SI Appendix, S2. d) Same as c, but volume between 2000 and 7000 Hz was obtained by matched filtering as described in SI Appendix, S2.

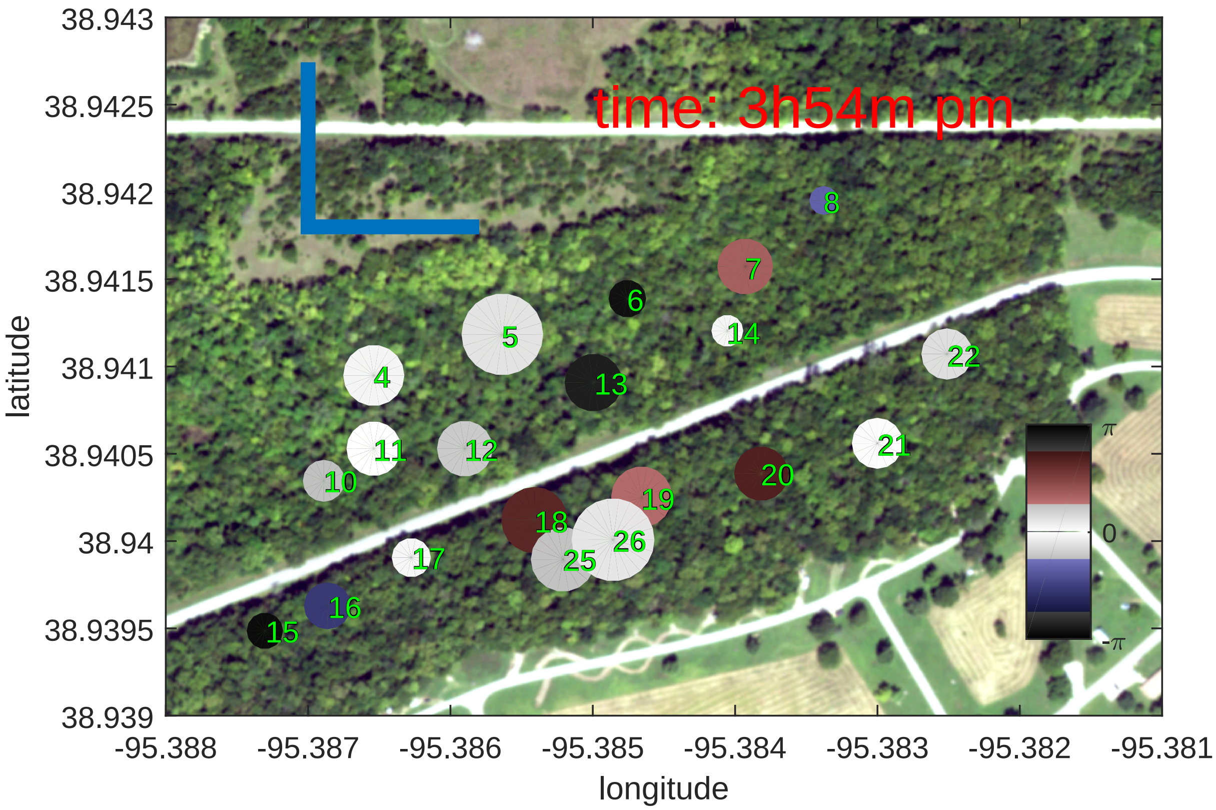

Fig. S22. Map of numbered measurement locations in space. The size of each circle marker represents that location's volume cycling amplitude at a particular point in time relative to its amplitude of cycling over all time. Color represents its phase relative to the phase of location 11 (a particularly synchronous location chosen as a reference) at this minute in time. The blue L shape is 100m on each edge. Aerial image data source: U.S. Department of Agriculture (USDA) Farm Service Agency's National Agriculture Imagery Program (NAIP) 2017 collection.

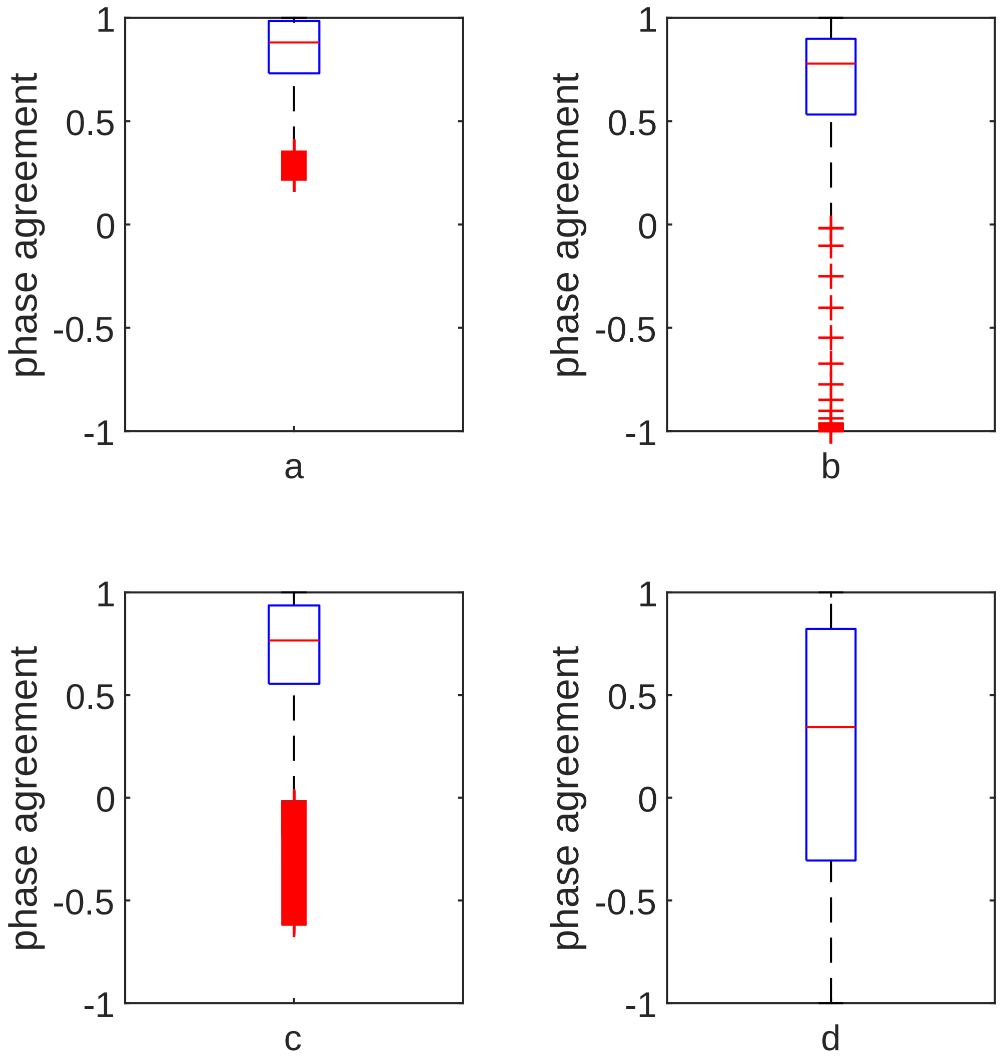

Fig. S23. Phase agreement of locations 4 and 11 is not due to ‘overhearing’; it is high when both locations exhibit strong oscillations. a) Phase agreement when both locations were oscillating strongly. b) Phase agreement when location 4 was oscillating strongly and location 11 was not. c) Phase agreement when location 11 was oscillating strongly and location 4 was not. d) Phase agreement when neither location was oscillating strongly. Methods and details in SI Appendix, S4.

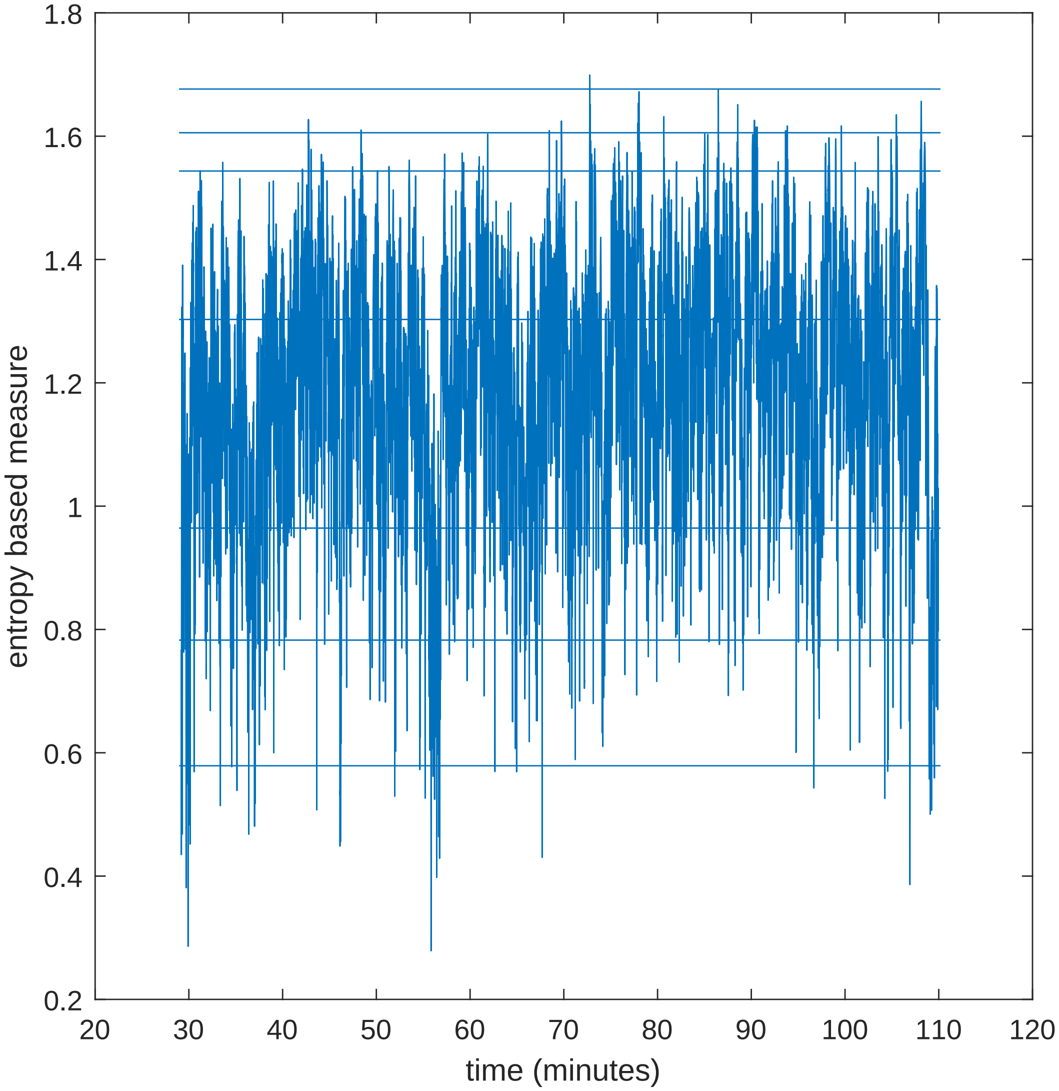

Fig. S24. Entropy of wavelet phases through time, plotted against 0.1, 1, 5, 50, 95, 99, 99.9 percentiles of entropy values obtained for random draws from a uniform phase distribution, with no smoothing. The percentiles are for the distribution of entropies of 20 independently uniformly distributed phases, corresponding a null hypothesis of independent cicada phases at the different recording locations. The empirical entropy is typically below the median (50th percentile, at approximately 1.3).

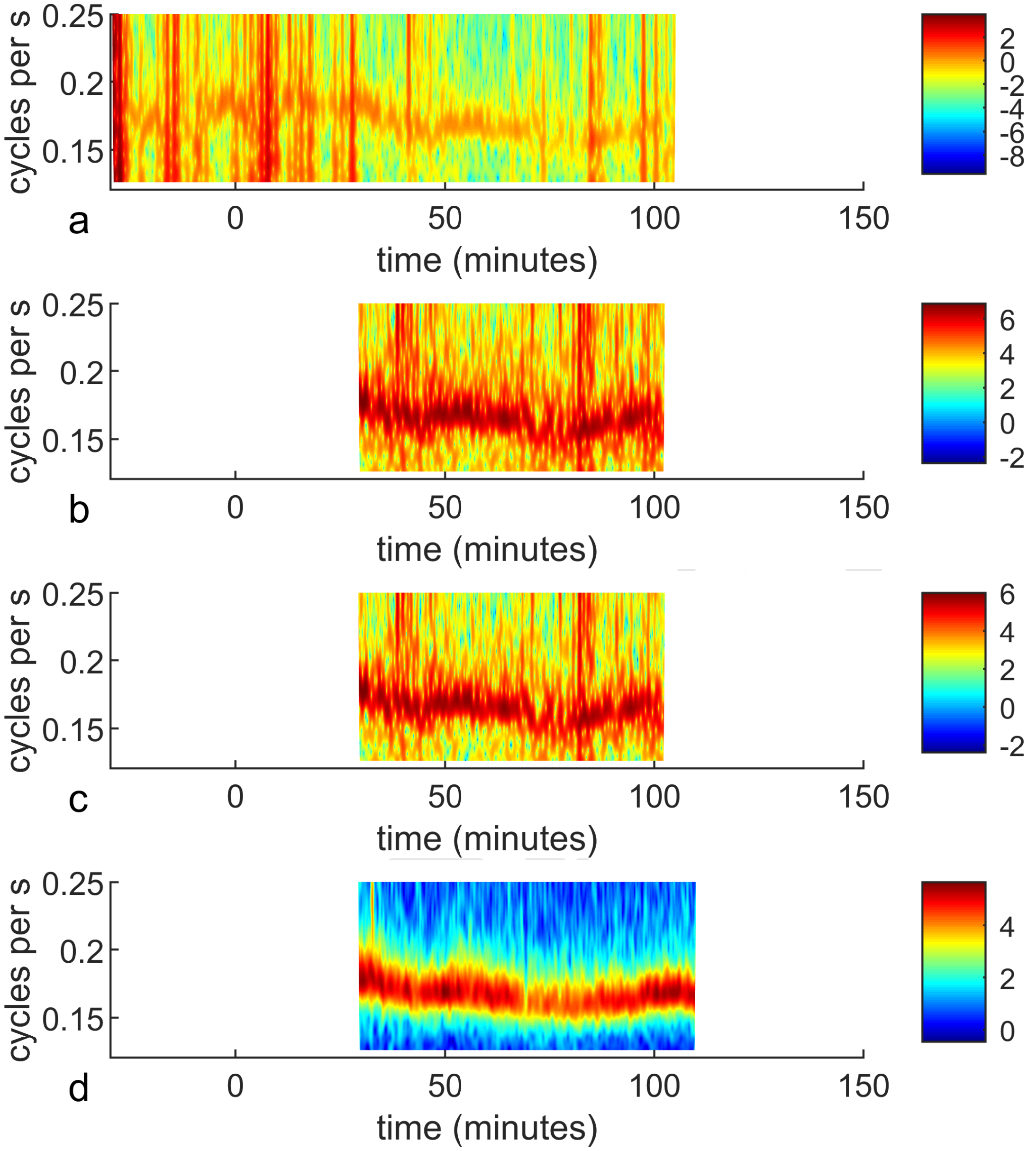

Fig. S25. Wavelet transform showing strong cycling of volume for long baseline reference recording device, varying around a period of approx five seconds. Note this recording ends before the end of the usable period of the other devices presented here, so transforms of two filtered versions of the signal are presented for all available data. Colour indicates log magnitude of fluctuation with a particular periodicity as this varies over time. a) Log magnitude of transform of volume in 0.1s windows through whole signal, starting from beginning of recording. b) Log magnitude of transform of available data: average FFT magnitude from 2000 to 7000 Hz (band-pass) in 0.1s windows starting from main pop. c) Log magnitude of transform of available data: average FFT magnitude mulplied by spectral filter in 0.1s windows starting from main pop. d) is the log magnitude of average transform of sites at the forest location for comparison, 3km away.

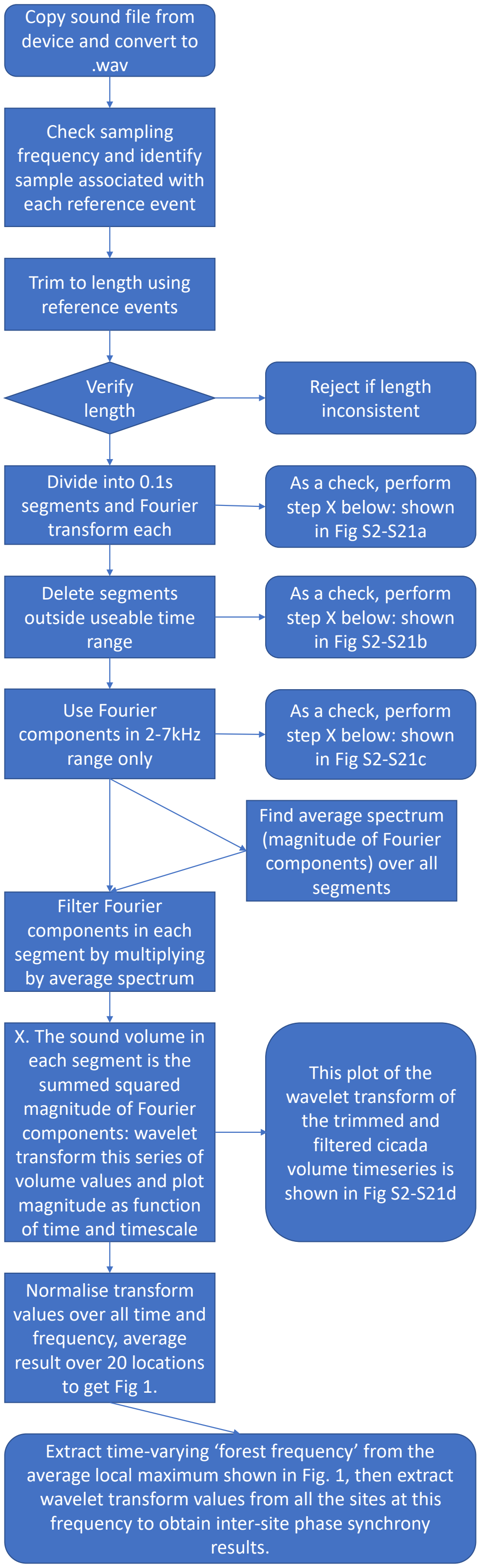

Fig. S26. Flow diagram showing steps in the processing of audio data (see Methods and SI Appendix, S2) before wavelet transforming the cicada sound volume time series.

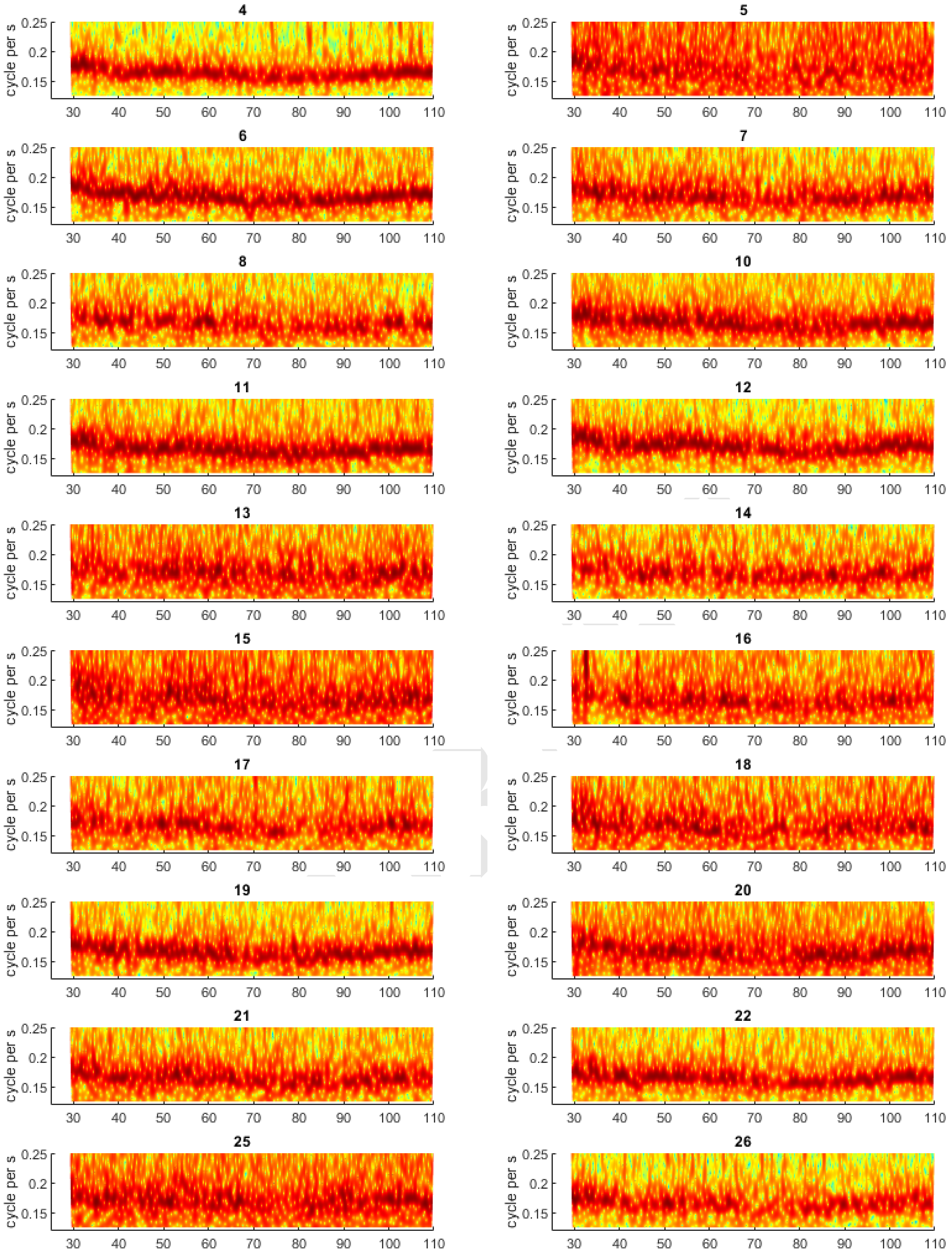

Fig. S27. Wavelet transform showing strong cycling of volume time series from all the recording devices on Fig. S22, with period varying around approximately five seconds. Each of these plots corresponds to panel d of a supplementary figure S2-S21, as identified by site number. Color indicates the log magnitude of volume fluctuations with a particular periodicity as this varies over time, volume between 2000 and 7000 Hz was obtained by matched filtering as described in SI Appendix, S2.

References

Addison, P.S. (2002) *The Illustrated Wavelet Transform Handbook: Introductory Theory and Applications in Science, Engineering, Medicine and Finance*. Taylor and Francis, New York.

Alatalo, R.V., Höglund, J. & Lundberg, A. (1991) Lekking in the black grouse- a test of male viability. *Nature* **352**, 155.

Alexander, R.D. & Moore, T.E. (1962) *The Evolutionary Relationships of 17-Year and 13-Year Cicadas, and Three New Species (Homoptera, Cicadidae, Magicicada)*, vol. 121 of *Miscellaneous Publications, Museum of Zoology, University of Michigan*. Ann Arbor.

Cooke, B.J., Nealis, V.G. & Régnière, J. (2007) Insect defoliators as periodic disturbances in northern forest ecosystems. *Plant disturbance ecology: the process and the response.* (eds. E. Johnson & K. Mlyamshl), Elsevier.

Cox, R.T. & Carlton, C.E. (1988) Paleoclimatic influences in the evolution of periodical cicadas (insecta: Homoptera: Cicadidae: Magicicada spp.). *The American Midland Naturalist* **120**, 183–193.

Duke, L., Steinkraus, D., English, J. & Smith, K. (2002) Infectivity of resting spores of massospora cicadina (entomophthorales: Entomophthoraceae), an entomopathogenic fungus of periodical cicadas (magicicada spp.) (homoptera: Cicadidae). *Journal of Invertebrate Pathology* **80**, 1–6.

Esper, J., Buntgen, U., Frank, D.C., Nievergelt, D. & Liebhold, A. (2007) 1200 years of regular outbreaks in alpine insects. *Proc. R. Soc. B* **274**, 671–679.

Fiske, P., Rintamaki, P.T. & Karvonen, E. (1998) Mating success in lekking males: a meta-analysis. *Behavioural Ecology* **9**, 328–338.

Fonseca, P.J. & Allen Revez, Æ.M. (2002) Temperature dependence of cicada songs (homoptera, cicadoidea). *J. Comp. Physiol. A* **187**, 971–976.

Gillooly, J. & Ophir, A. (2010) The energetic basis of acoustic communication. *Proceedings of the Royal Society B-Biological Sciences* **277**, 1325–1331.

Gogala, M. (2006) Acoustic website on european singing cicadas. *Slovenska Akademija Znanosti in Umetnosti Razred za Naravoslovne Vede Razprave* **47**, 155–164.

Goles, E., Schulz, O. & Markus, M. (2001) Prime number selection of cycles in a predator-prey model. *Complexity* **6**, 33–38.

Höglund, J. & Alato, R.V. (1995) *Leks*. Princeton Legacy Library.

Hoppensteadt, F.C. & Keller, J.B. (1976) Synchronisation of periodical cicada emergences. *Science* **194**, 335–337.

Johnson, D.M., Liebhold, A.M. & Bjornstad, O.N. (2006) Geographical variation in the periodicity of gypsy moth outbreaks. *Ecography* **29**, 367–374.

Kamata, N. (1998) Periodic outbreaks of the beech caterpillar, quadricalcarifera punctatella, and its population dynamics: the role of insect pathogens. *Proceedings: Population Dynamics, Impacts, and Integrated Management of Forest Defoliating Insects. USDA Forest Service General Technical Report NE-247.* (eds. M. McManus & A. Liebhold).

Koenig, W. & Liebhold, A. (2013) Avian predation pressure as a potential driver of periodical cicada cycle length. *The American Naturalist* **181**, 145–149.

Koenig, W.D. & Liebhold, A.M. (2003) Regional impacts of periodical cicadas on oak radial increment. *Can. J. For. Res.* **33**, 1084–1089.

Koenig, W.D., Ries, L., Olsen, V.B.K. & Liebhold, A.M. (2011) Avian predators are less abundant during periodical cicada emergences, but why? *Ecology* **92**, 784–790.

Kraskov, A., Stogbauer, H. & Grassberger, P. (2004) *Physical Review E* **60**, 066138.

Lloyd, M. & Dybas, H.S. (1966) The periodical cicada problem. ii. evolution. *Evolution* **20**, 466–505.

Luo, C., Wei, C. & Nansen, C. (2015) How do "mute" cicadas produce their calling songs? *PLoS ONE* **10**, e0118554.

Myers, J.G. (1929) *Insect Singers.* George Routledge and Sons, Ltd.

Nahirney, P., Forbes, J., Douglas, M., Chock, S. & Wang, K. (2006) What the buzz was all about: superfast song muscles rattle the tymbals of male periodical cicadas. *The FASEB Journal* **20**, 2017–2026.

DRAFT

Oberdorster, U. & Grant, P.R. (2007) Acoustic adaptations of periodical cicadas (Hemiptera : Magicicada). *Biological Journal of the Linnean Society* **90**, 15–24.

Pringle, J.W.S. (1953) Physiology of song in cicadas. *Nature* **172**, 248–249.

Schreiber, T. & Schmitz, A. (2000) Surrogate time series. *Physica D* **142**, 346–382.

Shelton, K.C.A.L. & Winkle, C. (2009) Effects of oviposition by periodical cicadas on tree growth. *Can. J. For. Res.* **39**, 1688–1697.

Singh, H., Mistra, N., Hnizdo, V., Fedorowicz, A. & Demchuk, E. (2003) Nearest neighbour estimates of entropy. *American Journal of Mathematical and Management Sciences* **23**, 301–321.

Singh, T. & Satyanarayana, J. (2009) Insect outbreaks and their management. *Integrated Pest Management: Innovation-Development Process* (eds. R. Peshin & A. Dhawan), Springer Science+Business Media B.V.

Storm, J.J. & Whitaker Jr., J.O. (2007) Food habits of mammals during an emergence of 17-year cicadas (hemiptera: Cicadidae: Magicicada spp.). *Proceedings of the Indiana Academy of Sciences* **116**, 196–199.

Sueur, J. & Sanborn, A.F. (2003) Ambient temperature and sound power of cicada calling songs (hemiptera: Cicadidae: Tibicina). *Physiological Entomology* **28**, 340–343.

Walker, T.J. (1983) Diel patterns of calling in nocturnal orthoptera. *Orthopteran Mating Systems: Sexual Competition in a Diverse Group of Insects* (eds. D.T. Gwynne & G.K. Morris), Boulder, CO: Westview.

Young, D. (1983) Pure-tone songs in cicadas with special reference to the genus magicicada. *Journal of comparative physiology. A, Neuroethology, sensory, neural, and behavioral physiology* **152**, 197–207.

Zilli, D., Parson, O., Merrett, G. & Rogers, A. (2014) A hidden markov model-based acoustic cicada detector for crowdsourced smartphone biodiversity monitoring. *Journal of Artificial Intelligence Research* **51**, 805–827.

DRAFT
